# From Central to Specialized Metabolism: An Overview of Some Secondary Compounds Derived From the Primary Metabolism for Their Role in Conferring Nutritional and Organoleptic Characteristics to Fruit

**DOI:** 10.3389/fpls.2019.00835

**Published:** 2019-06-28

**Authors:** Delphine M. Pott, Sonia Osorio, José G. Vallarino

**Affiliations:** Departamento de Biología Molecular y Bioquímica, Instituto de Hortofruticultura Subtropical y Mediterránea “La Mayora”, Universidad de Málaga – Consejo Superior de Investigaciones Científicas (IHSM-UMA-CSIC), Málaga, Spain

**Keywords:** quality traits, flavor, metabolic engineering, fruit, primary metabolism, secondary metabolism

## Abstract

Fruit flavor and nutritional characteristics are key quality traits and ones of the main factors influencing consumer preference. Central carbon metabolism, also known as primary metabolism, contributes to the synthesis of intermediate compounds that act as precursors for plant secondary metabolism. Specific and specialized metabolic pathways that evolved from primary metabolism play a key role in the plant’s interaction with its environment. In particular, secondary metabolites present in the fruit serve to increase its attractiveness to seed dispersers and to protect it against biotic and abiotic stresses. As a consequence, several important organoleptic characteristics, such as aroma, color, and fruit nutritional value, rely upon secondary metabolite content. Phenolic and terpenoid compounds are large and diverse classes of secondary metabolites that contribute to fruit quality and have their origin in primary metabolic pathways, while the delicate aroma of ripe fruits is formed by a unique combination of hundreds of volatiles that are derived from primary metabolites. In this review, we show that the manipulation of primary metabolism is a powerful tool to engineer quality traits in fruits, such as the phenolic, terpenoid, and volatile content. The enzymatic reactions responsible for the accumulation of primary precursors are bottlenecks in the transfer of metabolic flux from central to specialized metabolism and should be taken into account to increase the yield of the final products of the biosynthetic pathways. In addition, understanding the connection and regulation of the carbon flow between primary and secondary metabolism is a key factor for the development of fruit cultivars with enhanced organoleptic and nutritional traits.

## Introduction

Plant metabolism can be sub-divided into primary (or central) metabolism, which encompasses reactions and pathways absolutely vital for survival, and secondary (or specialized) metabolism, which fulfills a multitude of important functions for growth and development, including the interaction of the plant with the environment. Primary metabolism products derived from glycolysis, the TCA cycle, or the shikimate pathway often serve as precursors for the synthesis of the tens of thousands of secondary metabolites that have already been described (Kroymann, 2011). Compared to the differences of primary metabolism reactions, which are highly conserved, a much greater diversity is observed in secondary metabolism pathways at the level of species, organs, tissues, and cell level and even at different developmental stages (Wink, 2010). Furthermore, another factor that is presumably necessary for the large diversity of secondary metabolism is its high level of catalytic promiscuity, which is most likely due to its recent divergence from primary metabolism and the weaker selection pressure applied to secondary metabolic enzymes than to primary metabolic enzymes (Tokuriki et al., 2012). A recent metabolomic and statistical study comparing fruits from wild and domesticated accessions of strawberry showed that domestication caused the general dysregulation of secondary metabolism, while the core primary metabolites were maintained, suggesting the looser regulation of specialized metabolism (Vallarino et al., 2018). In addition, a correlation was observed between the taxonomic distribution of secondary metabolites and the gradual development of specialized tissue types and lifestyles in land plants (Weng, 2013). One example of this is the burst in the chemical diversity of volatile compounds to attract co-evolving insects concomitant with the rise of the angiosperms (Pichersky et al., 2006).

Several studies supported the evolution of secondary metabolism by the recruitment of enzymes and pathways from primary metabolism (de Kraker and Gershenzon, 2011; Kroymann, 2011; Carrington et al., 2018). Indeed, secondary metabolic pathways originate from different nodes of core primary metabolic pathway, suggesting that emergent enzymatic activities against primary metabolites yielded new compounds that were able to increase plant adaptation to particular environments and were gradually converted into specialized metabolites (Weng, 2013). It is thought that gene duplication, which is a very common key process in the plant kingdom for gain of new gene functions, is the mechanism by which specialized metabolism expanded to reach its current high level of diversity (Carrington et al., 2018). However, it is still unclear whether neofunctionalization followed gene duplication or gene duplication occurred as a consequence of an adaptive conflict present in the ancestral gene (Kroymann, 2011). In addition, it is interesting to note that new protein folds were not necessary for the emergence of specialized metabolism; on the contrary, gene families involved in secondary metabolism evolved with the use of primary metabolism protein folds (Weng et al., 2012).

de Kraker and Gershenzon (2011) and Carrington et al. (2018) presented two examples of enzyme recruitment from primary to secondary metabolism following gene duplication and the gain of a new function. The primary metabolite shikimate and the secondary metabolite quinate (which are structurally similar) are synthesized by shikimate and quinate dehydrogenases, respectively. Interestingly, both enzymes are members of the same gene family; however, due to a gene duplication event prior to the angiosperm/gymnosperm split, the two genes diverged into two different clades, allowing the evolution of quinate metabolism from primary metabolism (Carrington et al., 2018). An even more recent phylogenetic study confirmed that quinate dehydrogenases emerged from shikimate dehydrogenase sequences, and then evolved through independent gene duplication events in eudicots (Gritsunov et al., 2018). In addition, the authors demonstrated that very few changes in the amino acid sequence were necessary to modify the enzyme activity toward quinate synthesis.

Another striking example of the emergence of secondary metabolism emerged from central metabolism is the evolution of methylthioalkylmalate synthase (MAM), which catalyzes the committed step in the biosynthesis of precursors to glucosinolate, a secondary metabolite class involved in defense mechanisms in plants of the *Brassicaceae* family (de Kraker and Gershenzon, 2011). The MAM sequence is very close to that of isopropylmalate synthase (IPMS), which is involved in leucine synthesis. Phylogenetic studies indicated that the MAM enzyme most likely evolved from IPMS through gene duplication and a change in enzyme function. Once again, a few changes in the MAM sequence, specifically a deletion at the C-terminus, removed leucine-mediated feedback inhibition, and two amino acid changes in the catalytic sites were able to explain the recruitment of the enzyme from primary to secondary metabolic pathways.

## Secondary Metabolites in Fruit

By modifying central metabolite precursors, secondary metabolism is able to fulfill key functions involved in the interaction of the plant with its environment, particularly in relation to its biotic entourage. Three main classes of secondary metabolites are produced by plants: (1) terpenoid/isoprenoid, (2) phenolic, and (3) nitrogen/sulfur-containing compounds (Aharoni and Galili, 2011), which are produced from primary metabolism like TCA cycle, glycolysis, amino acids, pentose phosphate, and shikimate pathways. The fruit, an organ dedicated to seed protection and dispersal, has evolved in a multitude of forms that favors both its attractiveness to dispersers and its repellence to pathogens. Secondary metabolism in fruit carries out most of these functions by producing compounds involved in defense, pigmentation, and aroma (Leitzmann, 2016). In particular, polyphenol and terpenoid compounds are the main families of secondary metabolites produced by fruit during its growth and development (Poiroux-Gonord et al., 2010). Together with the presence of volatiles, polyphenol and terpenoid compounds are responsible for its unique aroma and its outstanding nutritional properties.

### Phenolic Compounds

Phenolic compounds or polyphenols are mostly produced through the shikimate and phenylpropanoid pathways. They are key contributors to the responses of the plants toward biotic and abiotic stresses, such as the protection against solar radiation and the robustness toward mechanical damage and in mediating defense against pathogens and herbivores. In addition, they are involved in flower and fruit pigmentation, important aspects for reproduction, and seed dispersal (La Camera et al., 2004; Vogt, 2010; Fraser and Chapple, 2011; Hassan and Mathesius, 2012). Due to their antioxidant and antiproliferative properties, they are highly valuable in human nutrition, and epidemiological studies suggest that a high dietary intake of polyphenols is associated with a decreased risk of cardiovascular and cancer diseases (Rodriguez-Mateos et al., 2014; Giampieri et al., 2015). Although polyphenols are a large and heterogeneous group of secondary metabolites, they are surprisingly derived from a very restricted set of basic structures whose origin is set in a primary metabolic pathway, the shikimate pathway (Herrmann, 1995). Polyphenolic compounds consist of multiple phenol ring backbones with hydroxyl groups or other substitutes like sugar molecules and organic acids (Manach, 2004). Several thousands of compounds with a polyphenol structure have been characterized in higher plants (Vogt, 2010).

**Figure 1:**
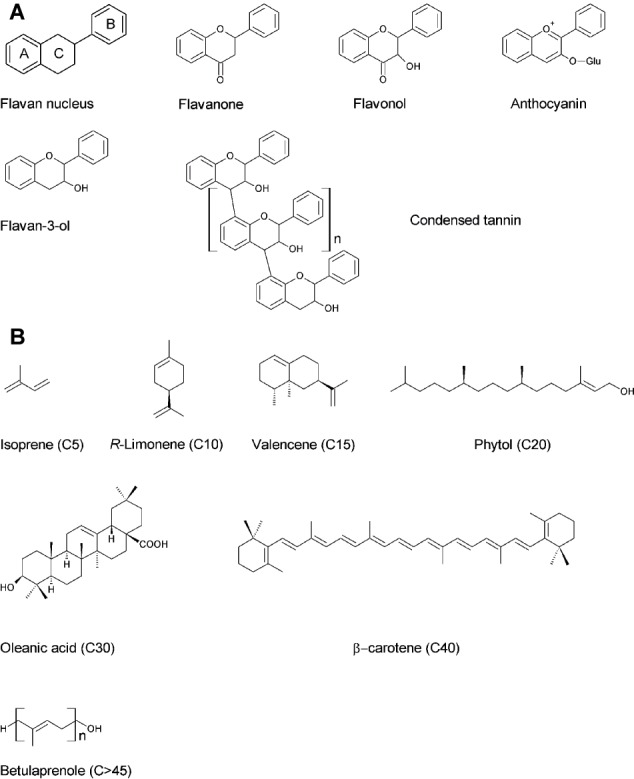
**(A)** Flavonoid chemical structures, including the flavan nucleus and the main classes of flavonoids found in fruits. **(B)** Terpenoid chemical structures, including isoprene and an example of mono-(R-limonene), sesqui-(valencene), di-(phytol), tri-(oleanic acid), tetra-(β-carotene), and polyterpenoid (betulaprenole) compounds.

Tannins are a group of polyphenols which can be divided into two classes: (1) condensed tannins (*syn*. proanthocyanidins) composed by flavan-3-ols polymers subunits linked *via* 4-6 and 4-8 interflavan bonds and (2) hydrolysable tannins that can be described as esters of gallic acid with a central polyol, typically β-D-glucopyranose (Ossipov et al., 2003). Glycosylation reactions of gallic acid yield penta-*O*-galloyl-β-D-glucopyranose requires particular attention because this derivative is the common precursor of all hydrolysable tannins. Further galloylation of penta-*O*-galloyl-β-D-glucopyranose gives rise to gallotannins, one of the two subclasses of hydrolysable tannins. Alternatively, penta-*O*-galloyl-β-D-glucopyranose can suffer oxidation reactions between different galloyl residues, forming 3,4,5,3′,4′,5′-hexahydroxydiphenoyl (HHDP) moieties, the core structure of the second subclass (ellagitannins) (Niemetz and Gross, 2005). Contrary to gallotannins, ellagitannins are widely spread in plant kingdom and form the largest group of known tannins. They are particularly abundant in berries of the Rosaceae family (strawberry and raspberry) and pomegranate. Hydrolysis of ellagitannins releases HHDP which spontaneously forms ellagic acid. Nowadays, tannins are intensively investigated because of their antioxidant, antimicrobial, antiviral, and antitumor characteristics (Landete, 2011). Two of the final products of the shikimate pathway, the aromatic amino acids phenylalanine and tyrosine, are phenylpropanoid precursors directed toward the secondary metabolism by the action of aromatic amino acid lyases, leading to the synthesis of both volatiles and non-volatile phenylpropanoids. In particular, phenylalanine ammonia lyase (PAL) catalyzes the deamination of phenylalanine to cinnamic acid and is the gateway enzyme to the phenylpropanoid pathway, directing carbon flow from primary to secondary metabolism (Vogt, 2010). The activities of several enzyme superfamilies (oxygenases, ligases, oxidoreductases, and transferases) are then responsible for the huge diversity of phenylpropanoids, being the formation of 4-coumaroyl CoA a decisive branch point within the pathway (Vogt, 2010). Phenylpropanoids range from simple phenolic acids, including derivatives of benzoic and cinnamic acids, to more complex compounds, such as stilbenes, lignans (lignin precursors), or the ubiquitous and well-studied flavonoids, which are present in many fruits. The shared structure of all flavonoids is the flavan nucleus, formed by A, B, and C rings, which are two aromatic rings (A and B) connected by three carbon atoms forming an oxygenated heterocycle (C). The distinct groups of flavonoids differ in the composition of their C heterocycle (Figure 1A; Hannum, 2004; Manach, 2004; Leitzmann, 2016). The flavonoid pathway starts with the formation of flavanones, which originate from the condensation of coumaroyl CoA and malonyl CoA molecules and are the first compounds with a flavan nucleus. Dihydroflavonols are then synthesized from flavanones and can be converted to anthocyanidins, which are colorless and unstable pigments. Anthocyanidin oxidation and glycosylation forms anthocyanins; these stable and colored compounds accumulate in vacuoles and confer a red, blue, pink, or purple color to many fruits, including eggplants, cherries, or strawberries (Petrussa et al., 2013). The reduction of anthocyanidins leads to the formation of flavan-3-ols, which are present in both monomeric (i.e., catechin) and oligomeric forms (condensed tannins). Indeed, the formation of condensed tannins occurs by the addition of anthocyanidin molecules to the terminal unit of flavan-3-ols (Bogs, 2005). Condensed tannins are present in many fruits, being responsible for their astringent flavor and preventing herbivore consumption when unripe (Manach, 2004).

A series of phenylalanine-derived volatiles with an aromatic ring include benzenoids, phenethyl compounds, phenylpropanes and phenylpropenes (Gonda et al., 2018). Phenylpropenes are present in many economically important fruits, such as tomato, strawberry, and grape, and originate from a side branch of the phenylpropanoid pathway. Indeed, their precursors are coniferyl and coumaryl alcohols, intermediates in lignin biosynthesis (Atkinson, 2018). The first committed step is the conversion of both alcohols to hydroxycinnamyl acetates, which are then reduced by phenylpropene reductases to produce volatiles such as eugenol, chavicol, and estragole, conferring aromatic spicy notes to the fruit (Araguez et al., 2013; Yauk et al., 2017). Other phenylalanine-derived volatiles, including methyl benzoate, benzyl acetate, and cinnamyl acetate, are synthesized from a lateral branch of the phenylpropanoid pathway, using PAL product cinnamic acid as precursor (Gonda et al., 2018). Finally, phenylalanine can produce phenethyl compounds in a PAL-independent manner, by the action of aromatic amino acid aminotransferase and decarboxylase (Tieman et al., 2006; Gonda et al., 2010; Atkinson, 2018).

### Terpenoid Compounds

The largest family of secondary metabolites identified to date belongs to the isoprenoid class, also known as terpenoids. Many terpenoids have a commercial interest since they are applied as pesticides, antimicrobial agents, and dietary anticarcinogenics. In addition, they are also used as precursors to produce chemicals, such as vitamins. All terpenoids derive from the mevalonate (MVA) pathway which is active in cytosol and starts from acetyl CoA, or from the methylerythritol-4-phosphate pathway (MEP), which is active in the plastids and starts from pyruvate and glyceraldehyde-3-phosphate (Rodríguez-Concepción and Boronat, 2002). Both pathways lead to the formation of the two 5-carbon isopentenoid building blocks: isopentenyl diphosphate (IPP) and its isomer, dimethylallyl diphosphate (DMAPP) (Laule et al., 2003). The MVA pathway produces IPP which is isomerized to DMAPP by isopentenyl diphosphate isomerase (IDI) (Hemmerlin et al., 2012). Isoprene, the smallest terpenoid with 5-carbon atoms, is produced from DMAPP (Behnke et al., 2007). Terpenoid larger than C5 are mono-(C10), sesqui-(C15), di-(C20), tri-(C30), tetra-(C40), and polyterpenes (>45) units (Figure 1B), that are formed by sequential head-to-tail condensation of IPP with DMAPP, geranyl diphosphate (GPP), farnesyl diphosphate (FPP), geranylgeranyl diphosphate (GGPP), and so on to increase the chain length (Jiang et al., 2016). These reactions are catalyzed by isoprenyl diphosphate synthases (IDS), also called prenyltransferases. In this review, we mainly focused on monoterpenes (C10), sesquiterpenes (C15), diterpenoid (C20), and carotenoids (C40) for their importance in the nutritional and aromatic quality of fruits.

#### Carotenoids

Carotenoids, a family of tetraterpenoid molecules, are widely distributed in plants, algae, fungi, and bacteria. In plants, carotenoids play a role as pigments, being responsible for the bright and appealing yellow, orange, and red tones of many fruits such as tomato, pumpkin, persimmon, and pepper (Sun et al., 2018). Furthermore, they play key roles in photosynthesis and photoprotection (Ruiz-Sola and Rodríguez-Concepción, 2012; Niyogi and Truong, 2013; Hashimoto et al., 2016), and they also provide precursors for the biosynthesis of the phytohormones, abscisic acids (ABA), and strigolactones (Nambara and Marion-Poll, 2005; Al-Babili and Bouwmeester, 2015). Moreover, they act as health-promoting phytonutrients and have been linked to the prevention of cardiovascular diseases, cancers, diabetes, Alzheimer’s, and other age-related diseases (Fraser and Bramley, 2004; Rao and Rao, 2007; Fiedor and Burda, 2014; Nuutinen, 2018).

Because of their important functional roles, significant efforts have been made to understand carotenoid metabolism in plants (Hirschberg, 2001; Fraser and Bramley, 2004; Botella-Pavía and Rodríguez-Concepción, 2006; Ruiz-Sola and Rodríguez-Concepción, 2012; Nisar et al., 2015; Liu et al., 2015b; Ikoma et al., 2016). Carotenoid biosynthesis pathway is well established. Geranylgeranyl diphosphate (GGPP; C20) is the direct carotenoid precursor, being formed by the condensation of three IPP and one DMAPP molecules. The first step of carotenoid biosynthesis is the condensation of two GGPP to produce phytoene (C40) that is catalyzed by phytoene synthase (PSY), which is the major rate limiting step (Cazzonelli and Pogson, 2010). Next, lycopene is formed from phytoene by a series of desaturation and isomerization reactions catalyzed by phytoene desaturase (PDS), ζ-carotene desaturase (ZDS), ζ-carotene isomerase (Z-ISO), and carotenoid isomerase (CRTISO). Cyclization of lycopene in which lycopene ε-cyclase and lycopene β-cyclase are involved gives rise to α-carotene and β-carotene (orange pigments), while following hydroxylation by two non-heme carotene hydroxylases (BCH1 and BCH2) and two heme hydroxylases (CYP97A and CYP97C) produces yellow xanthophylls (Sun et al., 2018). Oxidative cleavage by carotenoid cleavage dioxygenases (CCDs) and non-enzymatic cleavage of carotenoid molecules between the C9 and C10 position, yield to apocarotenoid formation (also called norisoprenes), including phytohormones and volatile compounds such as α- and β-ionone, 6-methyl-5-hepten-2-one, or geranylacetone that play an important role in the aroma of fruits like tomato, melon, or apricot (Beltran and Stange, 2016; Hou et al., 2016; Tieman et al., 2017; Wang et al., 2019). Despite the intensive research in this field, little is known about the regulation of carotenoid metabolism.

#### Volatile Terpenoids

Volatile terpenoids constitute the largest class of plant volatiles. Monoterpenes and sesquiterpenes play a key role in the interaction of the plant with its environment, being the most studied because of their broad distribution among angiosperms (Dudareva et al., 2004; Dudareva and Pichersky, 2008). These volatiles greatly contribute to floral emissions and the aroma of several fruits, including citrus, mango, grape, and strawberry (Hampel et al., 2006). As an example, the essential oil of *Citrus* fruits is mainly formed by the monoterpene *R*-limonene (Weiss, 1997; Feng et al., 2018), while *S*-linalool, which positively correlates with flavor intensity, is an important component of strawberry aroma (Aharoni et al., 2004; Yan et al., 2018). In addition, these volatiles play important roles in plant physiology such as signaling, attracting pollinators, and repelling or acting against predators and other leaf-damaging organisms (Loreto et al., 2014; Abbas et al., 2017). Recent studies demonstrated that emission of these volatiles by plants under biotic or abiotic stimuli, such as insect attacks or herbivore feeding, can lead to the transcriptional activation of defense genes in its neighbors (Tzin et al., 2015a; Richter et al., 2016; Markovic et al., 2019).

The pathway of volatile terpenoids biosynthesis can be summarized in three phases. As previously described, the first two phases are (1) the formation of IPP and DMAPP and (2) the sequential head-to-tail addition of IPP unit to DMAPP to form geranyl pyrophosphate (GPP), farnesyl diphosphate (FPP), and geranylgeranyl diphosphate (GGPP). The third phase of terpene volatile biosynthesis involves the conversion of the various prenyl diphosphates DMAPP, GPP, FPP, and GGPP to hemiterpenes, monoterpenes, sesquiterpenes and diterpenes, respectively, by the action of a large family of enzymes called terpene synthases (Bohlmann et al., 1998). Moreover, the huge diversity of volatile terpenoids is achieved through the action of terpene synthases, since they are able to generate multiple products from a single prenyl diphosphate precursor and many of them can also accept more than one substrate (Degenhardt et al., 2009; Bleeker et al., 2011). Another layer in volatile terpenoid diversification is formed by the action of enzymes through the transformation of the initial products by oxidation, dehydrogenation, and other reactions to increase their volatility and modulate their aromatic characteristics (Dudareva et al., 2013).

### Other Volatile Compounds

In addition to phenylpropanoid and terpenoid volatiles, primary metabolites, including carbohydrates, fatty acids, and amino acids, are also the direct precursors of many compounds that significantly contribute to the fruit aroma.

Substituted 4-hydroxy-3(2H)-furanones give caramel-like and sweet aromatic notes to some fruits, being particularly abundant in strawberry and pineapple, and are directly derived from carbohydrate metabolism. Indeed, D-fructose-1,6-diphosphate is the precursor of the 4-hydroxy-2,5-dimethyl-3(2)H-furanone, also known as furaneol. Methylation of furaneol produces 2,5-dimethyl-4-methoxy-3(2)H-furanone or mesifurane, which is another important component of the fruit aroma (Tokitomo et al., 2005; Raab et al., 2006).

Amino acid degradation is another important source of fruit volatiles. In particular, the catabolism of methionine, branched chain amino acids (leucine, isoleucine, and valine) and aromatic amino acids (phenylalanine, tyrosine, and tryptophan) yields a series of aldehydes, alcohols, and esters (Gonda et al., 2010).

The last steps of amino acid-derived volatile synthesis have been well studied in many fruits, being catalyzed by alcohol dehydrogenases (ADH, in which the aldehyde is reduced to the corresponding alcohol) and by alcohol aminotransferases (AATs), to form the corresponding esters (Pérez et al., 1996; Aharoni et al., 2000; Beekwilder et al., 2004; Manríquez et al., 2006; Yauk et al., 2017). However, the first steps of the pathway, upstream of ADH, have received less attention and are still not clearly defined (Tieman et al., 2006; Gonda et al., 2010; Kochevenko et al., 2012). Studies conducted in tomato and melon fruits using isotope labeling suggested that α-keto acids are key intermediates in the conversion of amino acids to volatiles and that, at least in the case of the branched-chain amino acids, they are probably more important precursors of the branched-chain volatiles than the amino acids themselves. The transamination of the amino acid to the corresponding α-keto acid occurs with the help of aminotransferase enzymes that reversibly catalyze the interconversion of amino acids and α-keto acids. In the second step, α-keto acids are decarboxylated to aldehydes, leading to the formation of volatiles (Gonda et al., 2010; Kochevenko et al., 2012).

Non-polar primary precursors, i.e., saturated and unsaturated fatty acids, also take part in generating plant volatiles, such as ketones, lactones, aldehydes, alcohols, and esters (Schwab et al., 2008). Indeed, free C18 unsaturated fatty acids, linoleic and linolenic acids, can be metabolized through the lipoxygenase (LOX) pathway, converting them into fatty acid hydroperoxides (HPOs). HPOs can be further converted by the action of fatty acid hydroperoxide lyases (HPLs), yielding aldehydes that can be reduced to their corresponding alcohols by the action of alcohol dehydrogenases. Alcohols generated through the LOX pathway can be further esterified by the action of AAT, forming straight chain esters (Rowan et al., 1999; Fellman et al., 2000; Li et al., 2014). These volatiles include C6 compounds, such as (Z)-3-hexenal, hexanal, hexanol, or hexyl acetate, known as “green leaf volatiles” due to their odor characteristics. Indeed, they confer the typical green, grassy and unripe notes of many fruits, and are released by plants under abiotic or biotic stress stimuli (Vogt et al., 2013; Mwenda and Matsui, 2014; Ul-Hassan et al., 2015; Vivaldo et al., 2017). In addition, the peroxidation of C18 polyunsaturated fatty acids by the LOX pathway is responsible for C5 volatile formation, in an HPL-independent manner (Shen et al., 2014). Together with “green leaf volatiles” and several C7, C8, and C10 volatiles, they are correlated with consumer preferences (Buttery et al., 1989; Hildebrand et al., 1989; Vogt et al., 2013; Mwenda and Matsui, 2014; Ul-Hassan et al., 2015).

The aroma of many ripe fruits is dominated by esters, which provide them with a fruity, sweet scent. Interestingly, the differences between aromatic and non-aromatic melon varieties lie in ester content. Indeed, both types produce amino acid-derived volatiles; however, in the aromatic varieties, these volatiles are normally esterified, while in non-volatile varieties, they are present as alcohols and aldehydes (Gonda et al., 2010). Fatty acids serve as precursors for straight chain esters, while branched chain esters originate from branched chain amino acids. In addition, aromatic esters (such as benzyl acetate) derive from phenylalanine (Wang et al., 2019).

## Metabolic Engineering for Fruit Quality Traits

For years of traditional breeding, farmers focused on yield, disease resistance, and fruit appearance. However, consumer preferences are currently forcing them to pay attention to fruit quality traits such as flavor and nutritional value (Tieman et al., 2017; Vallarino et al., 2019). For this reason, fruit metabolism has become an obvious target for the production of better-tasting and healthier fruits (Beauvoit et al., 2018). While primary metabolites, such as sugars and acids, directly influence fruit taste, secondary metabolites, such as polyphenols, terpenoids, and volatiles, are also responsible for their quality by being involved in their aroma, color, and health-promoting characteristics.

The modification of central metabolism in order to improve fruit aroma and nutrition by increasing the availability of precursor metabolites is an appealing idea; however, the regulation of primary metabolism is very tight, mainly because the plant needs to maintain a metabolic steady state. This is achieved by the multi-layered regulation of the involved enzymes and by the highly interconnected nature of the pathways, in which primary metabolic intermediates participate in several reactions (Sweetlove et al., 2017).

Secondary metabolic engineering for the (over)production of specialized metabolites is a much easier task, because the pathways are less interconnected than primary metabolism, and situated in a peripherical position of the network. Nevertheless, primary metabolism engineering for the accumulation of valuable secondary metabolites involved in fruit aroma and nutritional characteristics is a promising strategy, as it is reviewed here.

### The Shikimate Pathway and the Engineering of Phenylalanine Synthesis

**Figure 2:**
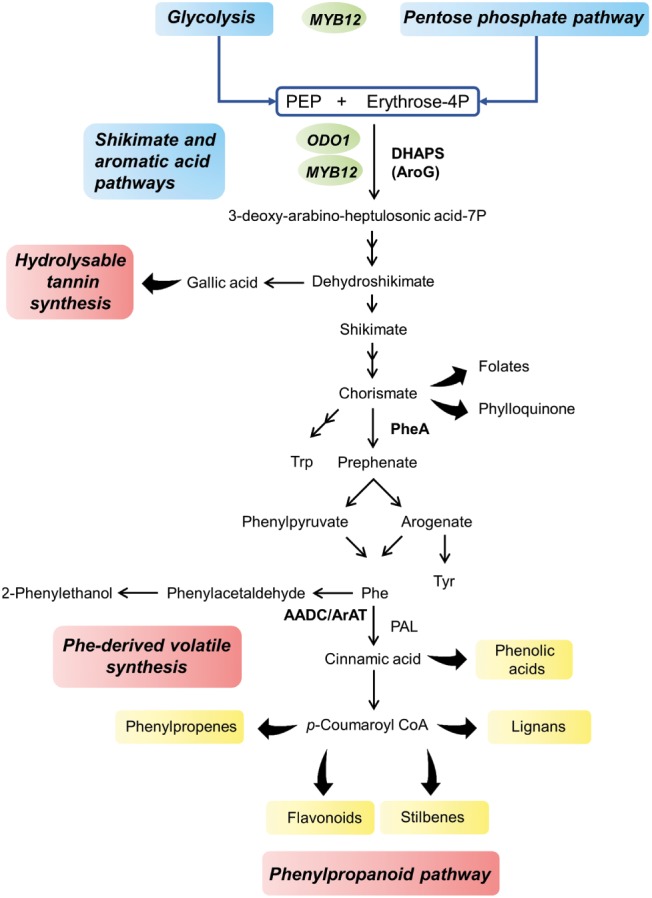
Overview of the shikimate pathway leading to the synthesis of phenolic acid compounds, including soluble (or hydrolysable) tannins, phenylpropanoids and phenylalanine-derived volatiles. Main classes of phenylpropanoids are shown in yellow. Primary pathways are indicated in blue, while secondary pathways are in red. Enzymes involved in the accumulation of primary metabolite precursors and possible targets for metabolic engineering are indicated in bold. Transcription factors involved in the regulation of carbon flux toward phenylpropanoid synthesis are shown in green (*ODO1* from *Petunia* and *MYB12* from *Arabidopsis*), and are located next to the pathways they controlled. PEP, phosphoenolpyruvate; DHAPS, 3-deoxy-d-arabino-heptulosonate 7-phosphate synthase; AroG, bacterial feedback insensitive DAHPS; PheA, bacterial feedback insensitive chorismate mutase/prephenate dehydratase; AADC, aromatic amino acid decarboxylase; ArAT, aromatic amino acid aminotransferase; PAL, phenylalanine ammonia lyase.

Phenylalanine, tyrosine, and tryptophan are synthesized through the primary metabolic shikimate pathway, being the first one the main aromatic amino acid produced (Figure 2); indeed, approximately 30% of the photosynthetically fixed carbon is directed through its synthesis to produce phenylpropanoids (Rippert and Matringe, 2002). For this reason, the shikimate pathway acts as a metabolic connection between central and specialized metabolism and a carbon flux checkpoint during the synthesis of secondary metabolites (Tzin et al., 2015b). In addition, bottlenecks in the conversion of primary metabolites into specialized metabolites must be identified to boost fruit aroma or the synthesis of health-promoting compounds. The transgenic expression of fundamental elements controlling a biosynthetic pathway can disturb the system and cause perturbations in metabolite accumulation, allowing the identification of bottlenecks in the process (Xie et al., 2016). Once identified, the genes (i.e., genes encoding enzymes or transcription factors) involved in the rate-limiting steps of the pathway can be converted into valuable tools for metabolic engineering. Such tools are available to study the synthesis of aromatic amino acids and their derivative secondary pathways are described below.

The first key enzyme of the shikimate pathway, 3-deoxy-D-arabino-heptulosonate 7-phosphate synthase (DAHPS), controls the amount of carbon entering the pathway, and converts phosphoenolpyruvate and erythrose-4-phosphate into 3-deoxy-D-arabino-heptulosonic acid 7-phosphate (Herrmann, 1995). Plants DAHPS enzymes are regulated by feedback inhibition loops (Graziana and Boudet, 1980; Reinink and Borstlap, 1982; Rubin and Jensen, 1985). Chimeric bacterial feedback-insensitive *DAHPS* genes have been suggested to inhibit the mechanism that promotes carbon flux toward the synthesis of aromatic amino acids and their specialized metabolite derivatives in *Arabidopsis* (Tzin et al., 2012), tomato (Tzin et al., 2013, 2015b; Xie et al., 2016), and red grape cv. Gamay cell suspensions (Manela et al., 2015).

Preliminary studies in the model plant *Arabidopsis* showed that the expression of bacterial feedback-insensitive DAHPS (*AroG* gene) increased the levels of shikimate, prephenate, and the aromatic amino acids phenylalanine and tryptophan compared to those observed in the absence of DAHPS expression. These results suggested that DAHPS enzymatically limits the flux of carbon from primary to secondary metabolism. Furthermore, the accumulation of the two aromatic acid precursor metabolites (shikimate and prephenate) indicated that the two downstream enzymatic steps are also possible bottlenecks in these pathways, at least in conditions in which DAHPS is not limiting (Tzin et al., 2012). *AroG* expression not only induced changes in central amino acid metabolism but also led to the accumulation of phenylalanine, which induced an increase of phenylalanine-derived secondary metabolites, such as lignin precursors, their derivatives, and flavonoids (Tzin et al., 2012). As many metabolites involved in fruit aroma and nutrition are phenylpropanoids and phenylalanine-derived volatiles (Tikunov, 2005), the regulatory effect of the DAHPS gene has been further studied in commercially important crops, (Tzin et al., 2013, 2015b). In addition, the phenylpropanoid pathway competes for the use of common precursors with terpenoids compounds (Negre-Zakharov et al., 2009). Alterations in primary metabolism were obvious in the fruit of tomatoes overexpressing the *AroG* gene under the *E8* promoter, a fruit-specific promoter that is induced by ethylene during fruit ripening (Tzin et al., 2013). As expected, the levels of shikimate and the three aromatic amino acids were increased, but alterations reached beyond the shikimate pathway, and the levels of several oligo- and monosaccharides, amino acids, and fumarate were altered compared to those in control tomato fruit. In addition, the modulation of secondary metabolism was also observed in fruits expressing the *AroG* transgene; on the one hand, the levels of several phenylpropanoids, both volatiles and non-volatiles, were increased in ripe fruits compared to those in control fruit, which was most likely due to the increase in the levels of their of primary precursors. On the other hand, the levels of carotenoids and volatile terpenoids decreased compared to those in control tomato fruit, confirming metabolic cross talk between the different primary and secondary pathways (Tzin et al., 2013). Indeed, the shift in metabolic flux toward oligosaccharides (*via* gluconeogenesis) and to the shikimate/phenylpropanoid pathways decreased precursor availability for terpenoid compounds.

Grape berries accumulate high levels of flavonoids, anthocyanins, benzenoids, and stilbenes. The ectopic overexpression of the *AroG* gene in a red grape cell suspension also led to the accumulation of phenylalanine and tyrosine (Manela et al., 2015). The levels of two specific polyphenols, the stilbene resveratrol and the flavonoid quercetin, increased by 20-fold and 150-fold, respectively, compared to those in control red grape cells as the consequence of phenylalanine accumulation. Interestingly, the authors confirmed that the increase in polyphenols was not the result of the enhanced expression of key genes of the stilbenes or phenylpropanoids pathways but was exclusively the result of increased phenylalanine content, suggesting that this amino acid is rate-limiting in the production of these metabolites. However, substrate availability probably does not control phenylpropanoid synthesis, as increased phenylalanine levels due to *AroG* expression did not lead to the accumulation of anthocyanins. Dihydroflavonol reductase, which is downstream of quercetin and upstream of anthocyanins in the flavonoid pathway, seemed to be unaffected by the increase in available substrate (Manela et al., 2015).

Another possible bottleneck in the synthesis of phenylalanine is the step catalyzed by the chorismate mutase/prephenate dehydratase, which converts chorismate into phenylpyruvate *via* prephenate. Following the same strategy, Tzin et al. (2009, 2015b) overexpressed a feedback-intensive bacterial form of the enzyme (*PheA* gene) in *Arabidopsis* and tomato fruit. In *Arabidopsis*, this led to an increase in phenylalanine levels compared to those in the control plants, which in turn modulated the secondary metabolite content; however, in tomato, *PheA* overexpression caused only insignificant changes (Tzin et al., 2015b). Interestingly, the ectopic co-expression of both the *AroG* and *PheA* genes produced a different metabolic profile than the one obtained with *AroG* expression alone. Levels of the phenylalanine-derived volatile phenylacetaldehyde were higher in the *AroG* and *PheA* co-expression lines than in the *AroG* lines, suggesting that additional regulatory mechanisms control the synthesis of metabolites derived from the shikimate and phenylalanine pathways. Phenylacetaldehyde contributes to the pleasant and fruity aroma of several fruits, such as tomato, grape, or plum, and is one of the most impactful compounds in persimmon aroma; as a consequence, its increase would be an interesting feature for quality trait breeding (Tieman et al., 2006; Pino and Quijano, 2012; Wang et al., 2012; Rambla et al., 2016). However, because of the cross talk between phenylpropanoid and terpenoid metabolism, the increase in phenylacetaldehyde in *AroG* and *PheA* co-expression lines was accompanied by a decrease in the levels of the terpenoid-derived volatiles β-ionone and geranylacetone, two compounds with a strong impact on overall fruit aroma appreciation (Zvi et al., 2012).

Due to the limitation in modulating the expression of enzymes controlling carbon flux from primary to secondary metabolism, the manipulation of transcription factors controlling a determined pathway appears to be a powerful tool for metabolic engineering.

Members of the R2R3-type MYB family act as regulators of aromatic amino acid biosynthesis and downstream secondary metabolites (Stracke et al., 2001; Liu et al., 2015a). The silencing of *ODO1*, a MYB factor in petunia required for the floral expression of *DAHPS* and other genes of the shikimate and phenylpropanoid pathways, caused a decrease in the levels of phenylalanine-derived volatiles compared to those in control plants (Verdonk, 2005).

The co-expression of *ODO1* and *AroG* in tomato fruit led to a higher phenylpropanoid content than that measured in either single transgene, probably for the combination of high substrate availability (higher phenylalanine content due to *AroG* expression) and the increased expression of key structural genes in the phenylpropanoid pathway (as a consequence of *ODO1* expression). *AroG-* and *ODO1-*expressing tomato fruits contained high levels of hydroxycinnamic acid derivatives, which act as antioxidants and antimicrobials (Korkina, 2007). Interestingly, both the volatile profile and the aromatic acid content were improved when compared to those in the single transgene plants, producing healthier and more appealing fruits (Xie et al., 2016).

*AtMYB12* is another MYB transcription factor that regulates flavonol synthesis in *Arabidopsis* (Luo et al., 2008). *AtMYB12* was overexpressed under the E8 promoter in tomato fruit, and resulted in 10% of fruit dry weight accumulation of flavonols and hydroxycinnamates (Zhang et al., 2015). Interestingly, this transcription factor was able to reprogram primary metabolism, driving carbon flux, ATP, and reducing power generated through central metabolism toward aromatic acid biosynthesis. This makes *AtMYB12* a great potential tool for engineering phenylpropanoid metabolism; indeed, Zhang et al. (2015) crossed *AtMYB12* with *Del/Ros1* tomato lines that accumulate high levels of anthocyanins in fruit (Butelli et al., 2008; Luo et al., 2008). Fruits resulting from the cross exhibited higher levels of chlorogenic acid, flavonols, and anthocyanins than either of the parental lines because of the activation of all the genes encoding primary metabolism enzymes related to flavonoid biosynthesis. Furthermore, *E8:AtMYB12* tomato lines can redirect metabolic flux toward the synthesis of the desired phenylpropanoids if the overexpression of the transcription factor is combined with a specific structural gene. As a proof of concept experiment, *E8: AtMYB12* co-expressing stilbene synthase from grape or isoflavone synthase from *Lotus japonicus* resulted in the highest yields reported to date of the stilbene resveratrol and the isoflavone genistein in tomato fruits (Zhang et al., 2015). As *AtMYB12* expression increases the aromatic amino acids content by the upstream activation of primary metabolism and by reprogramming carbon flux, this study opens the possibility of the manipulation of specialized metabolites derived from tyrosine and tryptophan in addition to the phenylpropanoids derived from phenylalanine.

In addition to serve as precursors for the synthesis of phenylpropanoids, the shikimate/aromatic amino acid pathway is also involved in the synthesis of essential micronutrients. In particular, phenylalanine-precursor chorismate is an important branch point within the pathway, leading to the synthesis of tetrahydrofolate (vitamin B9) (Wolak et al., 2017) and phylloquinone (vitamin K_1_) (Basset et al., 2017).

#### Folates

Folates are a group of water-soluble B vitamins, derivatives of tetrahydrofolic acid, which are synthesized only by plants and microorganisms. Folates are important components of the human diet, as they are needed for a large set of physiological processes. In particular, they play important roles in the biosynthesis of DNA (purines and thymidylate), but also for the production of methionine and vitamin B5 (Basset et al., 2004). It is known that folate deficiency can not only cause megaloblastic anemia and birth defects but also a low folate intake is associated with a higher risk to suffer cardiovascular disorders and several cancers (Ramírez Rivera et al., 2016). Folates are particularly abundant in some vegetables, legumes, and fruits (Hossain et al., 2004). In plants, folates are synthetized from pterin, glutamate, and *p*-aminobenzoate (*p*ABA) which derived from chrorismate (Díaz de la Garza et al., 2004). Strategies for folate biofortification have favored the engineering of the pteridine branch by acting upon the committed step. Indeed, this reaction, catalyzed by the GTP cyclohydrolase I (GCHI), seems to be rate-limiting as *GCHI* overexpression has led to significant folate increase in different crops, including rice and tomato (Díaz de la Garza et al., 2004; Hossain et al., 2004; Storozhenko et al., 2007). An alternative approach is the overexpression of the *aminodeoxychorismate synthase* (*ADCS*), the key enzyme of the *p*ABA pathway, catalyzing the synthesis of aminodeoxychorismate from chorismate. In rice, overexpression of both *GCHI* and *ADCS* led to a 100-fold folate increase (Storozhenko et al., 2007). Interestingly, Watanabe et al. (2017) showed that fertilization of hydroponically cultivated spinach with phenylalanine conduced to an increase in folate content. As both *p*ABA and phenylalanine are synthesized from chorismate, a phenylalanine excess could induce a negative feedback on the chorismate mutase or arogenate dehydratase, and so favor carbon flux toward *p*ABA synthesis (Watanabe et al., 2017). Phenylalanine fertilization of hydroponically grown crops, including fruits such as strawberries that are among the richest natural source of folates, seems an appealing and easy strategy, which in addition, avoids the use of genetically modified organism.

#### Phylloquinone

Phylloquinone (vitamin K1), a terpenoid-quinone conjugated component of the photosystem I, is an essential component in the human diet for its role in blood coagulation and bone metabolism (Saxena et al., 2001). The main dietary source of phylloquinone is green leafy vegetables; however, small amounts of phylloquinone are also found in fruits (Jäpelt and Jakobsen, 2016). Phylloquinone is a prenylated naphthoquinone which synthesis derived from two metabolic branches: (1) *via* chorismate the precursor of the naphthoquinone ring and (2) *via* MEP pathway for the formation of the phytyl diphosphate precursor (Lichtenthaler, 2010). Isochorismate synthase drives carbon flux from shikimate and chorismate pathway toward phylloquinone synthase, outlining the central role of chorismate in the synthesis of both primary and secondary metabolites (Verberne et al., 2007).

### Amino Acids Metabolism Engineering for Fruit Aroma

**Figure 3:**
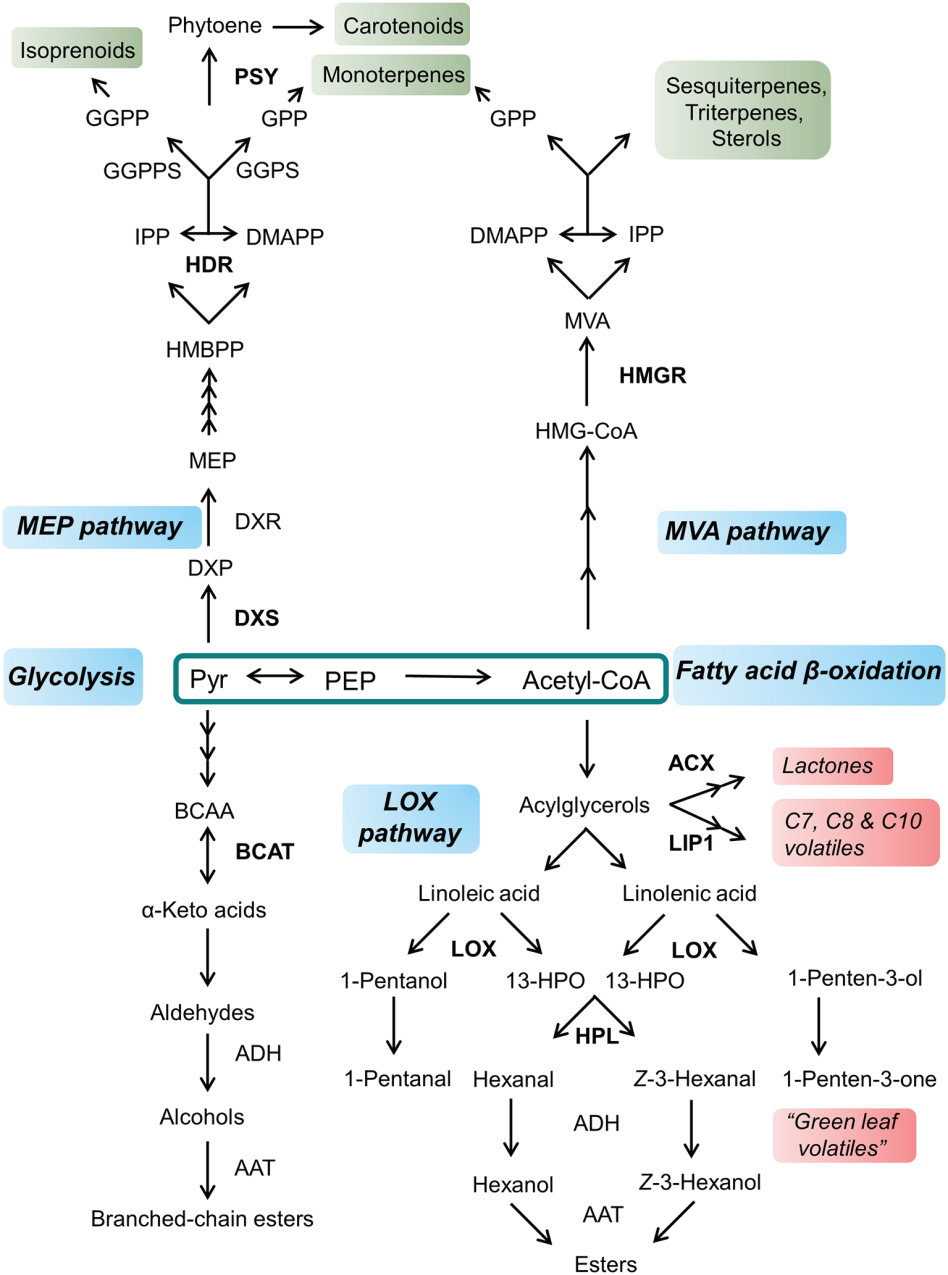
General overview of volatile and terpenoid compounds synthesis from pyruvate and acetyl-CoA. Enzymes involved in the accumulation of primary metabolite precursors and possible targets for metabolic engineering are indicated in bold. Primary pathways are indicated in blue; main classes of terpenoid compounds are shown in green, while volatile classes are emphasized in red. Pyr, pyruvate; PEP, phosphoenolpyruvate; BCAA, branched-chain amino acid; BCAT, branched-chain aminotransferase; ADH, alcohol dehydrogenase; AAT, alcohol aminotransferase; ACX, acyl-CoA oxidase; LIP1, lipase1; LOX, lipoxygenase; HPL, hydroperoxide lyase; DXS, 1-deoxy-D-xylulose 5-phosphate synthase; DXP, 1-deoxy-D-xylulose-5-phosphate; DXR, DXP reductoisomerase; MEP, 2-C-methyl-D-erythritol-4-phosphate; HMBPP, 1-hydroxy-2-methyl-2-(E)-butenyl 4-diphosphate; HDR, 1-hydroxy-2-methyl-2-(E)-butenyl 4-diphosphate reductase; IPP, isopentenyl diphosphate; DMAPP, dimethylallyl diphosphate; GPP, geranyl diphosphate: GGPS, GPP synthase; GGPP, geranylgeranyl diphosphate; GGPPS, geranylgeranyl diphosphate synthase; HMG-CoA, 3-hydroxy-3-methylglutaryl CoA; HMGR, 3-hydroxy-3-methylglutaryl CoA reductase; MVA, mevalonate.

Aroma is generated by a complex mixture of volatiles emitted by the fruit; however, even if hundreds of volatiles are detected in most fruits, a small subset of them is thought to be actually responsible for their distinctive fragrance (Jetti et al., 2007; Rowan et al., 2009; Klee and Tieman, 2018). Interestingly, these key volatiles are derived from a small set of primary metabolites, including phenylalanine, valine, leucine, isoleucine, methionine, and fatty acids (Figure 3; Klee and Tieman, 2018).

The first steps in amino acid-derived volatile synthesis have not been clearly established yet. Gonda et al. (2010) identified two aminotransferases, CmArAT1 and CmBCAT1, with aromatic amino acid (ArAT) and branched-chain amino acid aminotransferase (BCAT) activity, respectively, and able to convert amino acids into their respective α-keto acids. Their expression in melon fruit was similar to the pattern of accumulation of amino acid-derived volatiles and to the expression profiles of other known genes involved in volatile biosynthesis, such as AATs (Gonda et al., 2010; Li et al., 2016). In addition, climacteric melon cultivars exhibited higher *CmArAT1* and *CmBCAT1* expression levels than non-climacteric cultivars that accumulate fewer aroma-related compounds, supporting the role of both enzymes in volatile formation (Li et al., 2016). These results suggest that aminotransferases could be a suitable target in melon for metabolic engineering to favor carbon flux from primary precursors to volatile synthesis.

However, the overexpression of *BCAT1* (catabolic enzyme) and *BCAT3* (anabolic enzyme) in tomato plants showed minimal effects on the levels of branched-chain volatiles; even if an increase was observed compared to the levels of these volatiles in control plants, the effect was not large, indicating that further knowledge of the pathway is necessary to improve tomato fruit aroma (Kochevenko et al., 2012).

The lack of an effect on volatile synthesis observed in tomato overexpressing *BCAT1* and *BCAT3* is in accord with the absence of a correlation between the branched-chain amino acid pool and the expression levels of *BCAT1* and *BCAT3*, as described by Maloney et al. (2010). Additionally, there was no correlation between phenylalanine levels and the levels of its volatile derivatives (Tieman et al., 2006; Dal Cin et al., 2011). Rambla et al. (2016) did neither observe a significant correlation in different grape varieties between the abundance of branched-chain amino acids and the levels of their related volatiles, suggesting that this is a common characteristic of amino acids due to their involvement in a broad range of reactions and metabolic pathways. Taken together, these results indicate that amino acids, as central intermediates of cell metabolism, are most likely regulated by the multiple mechanisms, making it more difficult to use them as targets to improve aroma. Furthermore, it seems that the regulation of volatile synthesis occurs downstream of their precursor supply, at the level of α-keto acid precursors (Kochevenko et al., 2012). Indeed, Wang et al. (2019) showed that the decarboxylation of some α-keto acids by a pyruvate decarboxylase isoform (*PDC1*) highly expressed in melon fruit was a limiting step in the synthesis of ethyl and pentyl esters. In particular, the decarboxylation of 2-oxobutanoate by PDC1 leads to propanal synthesis, which is an intermediate in the formation of several straight chain esters (Wang et al., 2019).

Phenylalanine-derived volatiles, such as 2-phenylethanol, are also important for fruit flavor (Tieman et al., 2006). The formation of phenylalanine-derived volatiles is still not well understood and seems to differ between fruits from different species. In tomato, phenylalanine undergoes a two-step pathway, in which an initial decarboxylation reaction catalyzed by an aromatic amino acid decarboxylases (AADC) family member generates 2-phenethylamine, which is further deaminated to yield phenylacetaldehyde. The deamination reaction is probably carried out by an amine oxidase, dehydrogenase or transaminase, although the enzyme has not been identified. Finally, 2-phenylacetaldehyde reductase catalyzed the formation of 2-phenylethanol (Tieman et al., 2007). AADC activity seems to exercise major control over the metabolic flux from phenylalanine to multiple volatile compounds, which makes this pathway a good target for metabolic engineering (Tieman et al., 2006). Indeed, a tomato introgression line, IL-8-2-1, in which chromosome 8 was transferred from the wild tomato relative *Solanum pennellii* to the M82 cultivar, exhibited significantly higher AADC activity than the M82 tomato cultivar together with the increased emissions of 2-phenylacetaldehyde, 2-phenylethanol, 1-nitro-2-phenylethane, and 2-phenylacetonitrile and unchanged levels of free phenylalanine compared to those in the M82 tomato cultivar (Schauer et al., 2006). In addition, transgenic M82 plants overexpressing *AADC1A* or *AADC2* also showed increased levels of these volatiles compared to those in the M82 tomato cultivar (Tieman et al., 2006).

Using stable isotope feeding experiments in ripe melon fruit, Gonda et al. (2018) showed that phenylalanine is concomitantly metabolized by several biosynthetic pathways that operate in parallel, including both PAL-dependent and PAL-independent reactions. *CmArAT1* can convert phenylalanine to phenylpyruvate through a different pathway than that described in tomato (Gonda et al., 2010, 2018). In addition, both benzenoid and phenylpropanoid volatiles are generated through the action of PAL in the first committed step.

In addition to branched chain and aromatic aminotransferase activity, methionine aminotransferase activity was detected in melon fruit. Furthermore, exogenous methionine was able to increase the concentrations of sulfur-containing volatiles, which are an important aroma component of several fruits (Gonda et al., 2010, 2013). Using stable isotope-labeled precursors, Gonda et al. (2013) showed that two parallel pathways, involving a methionine aminotransferase and a methionine γ-lyase (MGL), respectively, were responsible for the degradation of methionine to sulfur volatiles. They confirmed that the second pathway was active in melon fruit by identifying *CmMGL*, a gene which expression increased along ripening and correlated with the presence of sulfur volatiles in different cultivars. More surprisingly, the degradation of methionine by MGL yielded isoleucine, suggesting a role of this amino acid in both sulfur and non-sulfur esters (Gonda et al., 2013).

While changes in the activity of the enzymes that transform amino acids to volatiles did not seem to have a strong effect on the precursor pools, another way of influencing it was suggested by Li et al. (2016). Indeed, they observed that melon treated with ethylene, which controls most ripening events in climacteric fruits, such as volatile biosynthesis (Giovannoni, 2004), showed increased levels of the vast majority of amino acids compared to those in melon without ethylene treatment. This result could indicate that not only ester formation but also the steps upstream of ester synthesis are under hormonal control. In contrast to the other cited studies, a positive correlation between phenylalanine content and most aromatic esters was observed, in addition to ethylene variation (Li et al., 2016).

### Fatty Acid Metabolism Engineering for Fruit Aroma

Lipoxygenases gene (*LOX*) family including non-heme, iron-containing dioxygenases ubiquitously present in plant. In plants, lipoxygenases are involved in several processes like seed germination, fruit ripening, and plant defense (Kessler et al., 2004; Yan et al., 2013). LOX catalyzes the oxidation of polyunsaturated fatty acids (linolenic acid, α-linolenic acid, and arachidonic acid) to form fatty acid hydroperoxides which serve as intermediates in the formation of physiologically active compounds such as oxylipins (Beaudoin and Rothstein, 1997). Depending on the positional specificity of fatty acid oxygenation, which can be C9 (9-LOX) or C13 (13-LOX) of the hydrocarbon backbone, two types of lipoxygenases are described (Feussner and Wasternack, 2002). Although many *LOX* genes have been identified in plants, only a reduced number of members have been described to be involved in aroma formation. As an example, 23 putative *LOX* genes were identified in apple; however only two, *MdLOX1a* and *MdLOX5e*, were associated with the production of green leaf volatiles by a QTL mapping (Vogt et al., 2013).

Tomato contains six *LOX*-encoding genes, named *TomloxA-F*, which can act on polyunsaturated fatty acids, at either the C9 or C13 position, yielding 9-hydroperoxides or 13-hydroperoxides (HPOs), respectively. Only 13-HPOs can be further metabolized into aromatic compounds *via* the action of hydroperoxide lyase (HPL), giving rise to C6 aldehydes (Chen et al., 2004). The *TomloxC* gene, which is highly expressed in ripening fruits, encodes a LOX enzyme essential for the generation of C5 and C6 aldehyde and alcohol volatiles, the most important contributors to consumer liking of fresh tomatoes (Shen et al., 2014). Silencing of the *TomloxC* gene led to a reduction in the level of both C6 and C5 fatty-acid-derived short-chain aldehydes and alcohols (Chen et al., 2004; Shen et al., 2014). However, the silencing of *HPL* resulted in a decrease in C6 volatiles and an increase in C5 volatiles compared to those in control plants, confirming that HPL is involved in the formation of C6 compounds, but not of C5 compounds (Shen et al., 2014). The higher emission of C5 volatiles in *HPL*-silenced plants than in control plants is most likely due to an increase in the content of 13-HPOs, which are driven toward C5 compound synthesis. This suggests that two separate LOX reactions first generate a hydroperoxide and then generate an alkoxyl radical that undergoes non-enzymatic cleavage to generate C5 alcohols (Shen et al., 2014). It would be interesting to see if the overexpression of *LOX* genes produced fruits with a better flavor, although there is no evidence that the activities of *LOX* isoforms are rate-limiting to volatile synthesis (Garbowicz et al., 2018). Zhang et al. (2017a) overexpressed a *13-LOX* gene from melon, *CmLOX18*, in tomato plants. Fruits from the transgenic lines showed enhanced emission of C6 volatiles together with an increased expression of *HPL* compared to the wild-type tomato plant. Interestingly, no changes in the expression of *Tomlox* genes or in the levels of C5 volatiles were observed, suggesting that *CmLOX18* is only involved in the synthesis of C6 volatiles in melon fruit (Zhang et al., 2017a). This result brought up the possibility of modulating *LOX* expression in the fruit to increase volatile emission and to determine whether this increase would correlate with better consumer acceptance.

The targeting of precursor content can be a valuable strategy to increase secondary metabolite and volatile levels, although, as previously described in this review, this approach remains limited, possibly because metabolic intermediates have a stronger influence on the final product of the pathway than the primary metabolite precursors (Gonda et al., 2010; Qin et al., 2014). The *in vitro* incubation of kiwi, melon, or tomato fruits with fatty acid precursor linoleic and linolenic acids increased the emission of C6 aldehydes, alcohols, and straight chain esters compared to that of the control fruit (Zhang et al., 2009; Ties and Barringer, 2012; Tang et al., 2015). Similar results were obtained in pear fruit; however, ester increase was more noticeable when the fruits were incubated with the direct C6 volatile precursors hexanol and hexanal (Qin et al., 2014). Contreras et al. (2016) monitored ripening-dependent changes in the pool of free fatty acids and volatiles in apple and observed an increase in the levels of hexanol, hexanal, and esters containing hexyl moieties derived from hexanol concomitant with the increase of linoleic acid over time. In addition, the absence of linolenic acid detection in the Jonagold apple cultivar was linked with a deficiency in the emission of *cis*-3-hexenal, *cis*-3-hexenol, and *cis*-3-hexenyl acetate, suggesting that the availability of the precursor fatty acid is a key element in the fruit aroma pattern (Contreras et al., 2016). Taken together, these results suggest that free fatty acid content is a good candidate for metabolic engineering. Furthermore, the regulation of fatty acid catabolism through the LOX pathway by phytohormones is another interesting factor to take into account for the accumulation of valuable aromatic compounds. Indeed, a study in grape cell culture suggested that ABA and methyl jasmonate have a positive effect on LOX activity, leading to a mayor accumulation of C6 and C9 volatiles (Ju et al., 2016).

To date, very little is known about the initial steps leading to fatty acid-derived volatile biosynthesis. The first step would likely include the release of polyunsaturated fatty acids from mono-, di-, and triglycerides by the action of a lipase. Very recently, a QTL analysis of introgression lines from the wild tomato *S. pennellii* into the cultivated species *S. lycopersicum* (Eshed and Zamir, 1995) allowed the identification of a class III lipase, LIP1, involved in the degradation of triacylglycerols into glycerol and free fatty acids in fruits (Garbowicz et al., 2018). The introgression line, IL 12-3, and backcrossed introgression lines (BILs) containing introgressions from *S. pennellii* that overlap those that of IL 12-3 contained a *S. pennellii* genome region that was correlated with significantly lower levels of diacylglycerols and triacylglycerols and higher levels of six fatty acid-derived volatiles, including several C5, C10 (*Z*-4-decenal), and C12 (*E,E*-2,4-decadienal) aldehydes, than those in control plants. Fine QTL mapping, using a sub-ILs population derived from a cross between *S. pennellii* IL 12-3 and M82 *S. lycopersicum* confirmed that the *S. pennellii* locus co-localized with the *LIP1* locus. In ripening fruits, *SpLIP1* transcripts accumulate to several thousand-fold higher levels than those in its *S. lycopersicum* ortholog, possibly due to an insertion into the *SlLIP1* promoter region. In addition, a positive correlation between *SpLIP1* transcript abundance and the emissions of C10 aldehydes was observed, suggesting that the content of these volatiles is controlled by *SpLIP1* expression.

The silencing of *SpLIP1* in IL 12-3 led to an increase in triacylglycerols and a reduction of C5-C10 volatiles, confirming the role of LIP1 in generating free fatty acid precursors for aroma compound synthesis. As expected, the overexpression of *SpLIP1* in the M82 cultivar decreased the levels of diacylglycerols and triacylglycerols and increased the glycerol content compared to those in the control M82 cultivar. However, no significant increase in the emissions of fatty acid-derived volatiles was observed, which was probably because of the initial position of *LIP1* in the metabolic pathway and its limited effect on the overall flux rate. Nevertheless, introducing the *SpLIP1* allele into elite tomato varieties would allow the specific increase in the content of multiple volatiles that are positively correlated with consumer preference and holds promise for the metabolic engineering of fruit aroma (Garbowicz et al., 2018).

Fatty acids are also precursors for the synthesis of lactones and furanones, which are important molecules that contribute to the aromas of fruits such as peach or mango (Deshpande et al., 2017; Zhang et al., 2017b). Several studies have demonstrated that the β-oxidation of fatty acids in the peroxisome is one of the main lactone biosynthesis pathways, as a positive correlation was found between lactone content and acyl-CoA oxidase (ACX) activity in peach (Zhang et al., 2017b). ACX catalyzes the first step in the fatty acid β-oxidation and is rate-limiting in the biosynthesis of lactone volatiles. Specifically, a high correlation was found between γ-decalactone accumulation, ACX activity against C_16_-CoA substrate and *PpACX1* content in the mesocarps of ripe peaches (Zhang et al., 2017b).

In mango fruit, the lipoxygenase *Mi9LOX* utilizes linoleic and linolenic acids as substrates, and the overexpression of this gene induced a significant increase in the concentration of δ-valerolactone and δ-decalactone compared to that in control mango fruit. In addition, the overexpression of *MiEH2*, which encodes an epoxide hydrolase involved in lactone synthesis that catalyzes the hydrolysis of fatty acid epoxides (Haffner and Tressl, 1998), yielded higher levels of δ-valerolactone, δ-hexalactone, and γ-hexalactone than those observed in control fruit (Deshpande et al., 2017).

*Mi9LOX* and *MiEH2* are part of the lipoxygenase and monooxygenase pathways respectively. As previously described in this section, HPOs generated through the lipoxygenase pathway can be redirected to the formation of C6 aldehydes and ketones *via* the *HPL* pathway. In addition, Deshpande et al. (2017) suggested that HPOs can also serve as substrates for the peroxygenase pathway, producing epoxy and monohydroxy fatty acids and leading to the formation of lactones. Consequently, lactone synthesis in mango competes with HPL pathway for the use of common precursors.

### The Engineering of Primary Terpenoid Precursors for Carotenoid and Monoterpene Content in Fruit

Generally, the MEP pathway supplies precursors to monoterpenes, diterpenes, and carotenoids, while the MVA pathway provides building blocks for sesquiterpene, triterpene, and sterol synthesis, although cross talk and exchange occurs between the metabolites of both pathways (Figure 3; Laule et al., 2003; Eisenreich et al., 2004; Gutensohn et al., 2013). Several studies have noted that the metabolic flux through the MEP pathway is often higher than through the MVA pathway, and the export of plastidial IPP plays an important role in cytosolic terpene synthesis in some plants (Dudareva et al., 2005; Wu et al., 2006; Orlova et al., 2009). It is thought that the metabolic flux in the MVA pathway is controlled by the 3-hydroxy-3-methylglutaryl CoA reductase (HMGR), which catalyzes the formation of MVA from 3-hydroxy-3-methylglutaryl CoA. Indeed, overexpressing a *HMGR* gene from *Arabidopsis* in tomato plants conduced to an increase of phytosterol content in ripe fruits (Enfissi et al., 2005). In addition to phytosterols, the reaction synthesized by HMGR seems to be also the rate-limiting step in triterpene synthesis. Overexpression of the gene in *Platycodon grandiflorum*, a medicinal plant well known in Asia, led to enhanced levels of platycodins, a group of triterpene glycosides which are the main pharmacological components present in the roots (Kim et al., 2013).

Two primary metabolites, pyruvate and glyceraldehyde-3-phosphate, derived from glycolysis and the pentose phosphate pathway are the substrates of the first enzyme of the MEP pathway, 1-deoxy-D-xylulose 5-phosphate synthase (DXS). DXS catalyzes the condensation of pyruvate and glyceraldehyde-3-phosphate, producing 1-deoxy-D-xylulose-5-phosphate (DXP), which is then isomerized to MEP by DXP reductoisomerase (DXR). Five other steps lead to the formation of two terpene precursors, IPP and DMAPP, with 1-hydroxy-2-methyl-2-(E)-butenyl 4-diphosphate reductase (HMBPP reductase or HDR) as the last enzyme in the pathway (Saladié et al., 2014). The MEP pathway is tightly regulated at multiple levels, although studies in different plant species have noted that the control of the pathway is exerted mainly by DXS, both at the transcriptional level and in term of protein abundance and activity (Lois et al., 2000; Wright et al., 2014; Simpson et al., 2016).

The products of the MEP pathway, IPP and DMAPP, then condense to form GGPP in a reaction catalyzed by the GGPP synthase (GGPPS). As several genes encoding putative GGPPS have been identified in most plant genomes, it is likely that different isozymes could be involved in the synthesis of each specific group of isoprenoids (Lange and Ghassemian, 2003).

The MEP pathway and carotenoid biosynthesis are coordinated at the gene expression level, as the expression of the genes involved in the first pathway precedes or correlates with the expression of genes in the second one (Bouvier et al., 1998; Lois et al., 2000; Botella-Pavía et al., 2004). Regulation and cross talk between both pathways have been previously reviewed by Rodríguez-Concepción (2010) and will not be discussed here in detail. However, a few key steps seem to control metabolic flux from primary to secondary metabolism and will be summarized in the next section.

In tomato, *LeDXS1* is responsible for the accumulation of carotenoid pigments during fruit ripening (Lois et al., 2000). Indeed, an increase in *LeDXS1* expression was concomitant with the largest accumulation of carotenoid at the orange fruit stage as observed in other fruits such as pepper (Bouvier et al., 1998). During ripening, a shift between lutein and β-carotene, which accumulate at the green stage, and lycopene, which is the main carotenoid at the red stage, occurs (Ronen et al., 1999). Significant cross talk occurs between the MEP and carotenoid pathways that may contribute to the regulation of pigment accumulation in the fruit, as the expression of *DXS* correlates with the transcript abundance of the committed enzyme of carotenoid biosynthesis, a fruit-specific phytoene synthase, *PSY1.* However, *PSY1* transcripts start to accumulate before *DXS* transcripts accumulation and the increase in carotenoid content, suggesting that the synthesis of the precursor, DXP, is the rate-limiting step in lycopene synthesis (Lois et al., 2000). In addition, the injection of 1-deoxy-D-xylose, which can enter the MEP pathway, into mature green tomatoes upregulated *PSY1* expression compared to that in control tomatoes, confirming the regulatory role of *DXS* in the carotenoid pathway (Lois et al., 2000). In orange (*Citrus sinensis*) juice sacs, carotenoid synthesis is also controlled by *DXS* and *PSY1* in a coordinated way during fruit ripening, leading to a general increase in the biosynthetic genes of the carotenoid pathway (Alós et al., 2006; Fanciullino et al., 2008).

In addition to DXS, the last enzyme of the MEP pathway, HDR, may play an important role in the regulation of plastidial isoprenoid precursors for carotenoid biosynthesis (Botella-Pavía et al., 2004; Vranová et al., 2012). Indeed, the increased expression of *HDR* was also observed simultaneously with carotenoid accumulation in ripening tomatoes. It is possible that the coordinated upregulation of *DXS, HDR*, and *PSY* transcript levels is necessary to drive metabolic flux toward carotenoid synthesis (Botella-Pavía et al., 2004).

Taking advantage of the regulatory function of *DXS*, Enfissi et al. (2005) showed that the overexpression of an *E. coli DXS* in tomato plants increased DXS enzyme activity and carotenoid content compared to those in control tomato plants, even though the accumulation of the final product, carotenoids, was disparate. Indeed, the phytoene content was strongly increased compared to that in control tomato plants, suggesting that phytoene desaturation is the limiting step in the absence of DXS bottleneck. This is yet another example of the challenge in precursor metabolite engineering; elevated *DXS* expression was only able to influence the pathway when adequate precursors from intermediary metabolism were accessible (Enfissi et al., 2005).

In tomato and *Citrus*, carotenoid formation during fruit ripening is dependent on type I DXS (Paetzold et al., 2010; Peng et al., 2013). In melon, however, two type II DXS enzymes (CmDXS2a and CmDXS2b) are the principal isoforms in the fruits of orange varieties and are probably responsible for plastidial isoprenoid accumulation. In varieties that lack carotenoid pigments, such as “Piel de Sapo,” neither of these CmDXS2 enzymes were upregulated, implying that only varieties that contain high levels of carotenoids require elevated DXS activity (Saladié et al., 2014).

Another study in clementine pointed out the connection between carbohydrate metabolism and the accumulation of carotenoids. Indeed, they saw that sugar starvation at the early stages of fruit development led to an increase in carotenoid content. In this case, the relation between primary and specialized metabolism would be linked to plastid capacity in accumulating carotenoids, and not to precursor availability (Poiroux-Gonord et al., 2013).

The IPP and DMAPP produced through the MEP pathway can condense to form GPP with the help of GGP synthase (GGPS). GPP then serves as a substrate for monoterpene synthase, which catalyzes the branch point step in monoterpene biosynthesis (Gutensohn et al., 2013). GGPPS, which is involved in carotenoid synthesis, is influenced by heterodimeric GGPSs, which contain a small subunit able to interact with GGPPS and to change its product specificity from GGPP to GPP (Tholl et al., 2015). Based on this knowledge, tomato plants were produced that overexpress the small subunit of a heterodimeric *GGPS* (*GGPS-SSU*) from snapdragon under the control of a fruit-specific promoter (Gutensohn et al., 2013). The massive metabolic flux of the MEP pathway, which was induced during tomato ripening to support carotenoid accumulation, was driven toward monoterpene synthesis, and volatiles were almost not detected in non-transformed plants due to the low expression of monoterpene synthase in ripening tomato fruits (Buttery et al., 1989; Falara et al., 2011; Gutensohn et al., 2013). Even the monoterpene content was increased when geraniol (monoterpene) synthase was overexpressed together with *GGPS-SSU* (Gutensohn et al., 2013).

Other studies in grape correlate DXS activity with monoterpene content (Battilana et al., 2009; Duchêne et al., 2009; Emanuelli et al., 2010; Dalla Costa et al., 2018). Indeed, the well-appreciated floral flavor of Muscat varieties is associated with the presence of monoterpenoids, such as linalool, geraniol, nerol, citronellol, and α-terpineol (Ribéreau-Gayon et al., 1975). Interestingly, a major QTL for monoterpene production in Muscat varieties co-localized with *VvDXS1* (Battilana et al., 2009; Duchêne et al., 2009). Emanuelli et al. (2010) assessed the association between nucleotide variation in the *DXS* sequence and Muscat flavor, and found a non-synonymous amino acid difference in over 95% of the Muscat-flavored genotypes studied.

Dalla Costa et al. (2018) confirmed by analyzing a grapevine germplasm collection that the nucleotide changes in the sequence of *VSDXS1* were able to explain the differential accumulation of monoterpenes in Muscat and non-Muscat grape varieties. In addition, this genetic variation seemed to also modulate sesquiterpene content. Wild-type Muscat *VvDXS1* (with increased catalytic efficiency) alleles were overexpressed in the “microvine,”, “Chardonnay,” and “Brachetto” cultivars. At flowering, the transgenic plants showed the upregulation of the *HDR* gene and of three genes of the MVA pathway compared to those of the control plants, suggesting that both pathways could be more deeply integrated than previously thought (Chaurasiya et al., 2012; Dalla Costa et al., 2018). In addition, genes from the downstream carotenoid, monoterpene, and sesquiterpene pathways were slightly upregulated compared to those of the control plants. During veraison and at grape maturity, most of the MEP and MVA pathway genes were upregulated, together with the genes involved in the first steps of the carotenoid pathway compared to those of the control plants, while at maturity, few changes were observed between the transgenic plants and the control plants. When the fruits were ready to harvest, the monoterpene content was significantly higher in the transgenic plants than in the control plants, and the increase was more pronounced in the plants overexpressing the Muscat allele, most likely due to its improved catalytic performance (Dalla Costa et al., 2018). This is another example of the ectopic expression of *DXS1* supporting metabolic flux through the MEP pathway to improve the volatile content of the fruit. Additionally, it was possible to increase final metabolites levels by searching for alleles with better catalytic efficiency.

### Engineering for Tocopherol Content

Tocopherols are lipid-soluble antioxidants that are exclusively synthesized by photosynthetic organisms. They have a high nutritional value, as several of them show vitamin E activity (Pryor et al., 2000). These compounds contain a chromanol group derived from homogentisate and an isoprenoid-derived chain resulting from the MEP pathway. Homogentisate is derived from the shikimate pathway, where its formation is catalyzed by 4-hydroxyphenylpyruvate dioxygenase (HPPD) in the first step of the tocopherol-core pathway. *HPPD* expression increased along with the accumulation of tocopherol and carotenoid levels during mango fruit ripening (Singh et al., 2011). However, neither *HPPD* nor *DXS* overexpression alone in tomato was sufficient to redirect the flux toward tocopherol synthesis. The expression of another gene, *geranylgeranyl reductase (GGDR)*, which is responsible for the availability of the ultimate tocopherol precursor, phytyl-diphosphate, was correlated with an increase in vitamin E content in mature green tomato fruit and was a bottleneck for the isoprenoid precursor availability (Quadrana et al., 2013). In addition, an analysis of different tomato genotypes noted that the genotypes containing high-tocopherol levels presented attenuated carotenoid levels, suggesting that precursor competition is the main limiting factor for vitamin E synthesis (Quadrana et al., 2013). As a consequence, metabolic engineering for enhanced tocopherol content in fruit should include the manipulation of both structural genes from the core tocopherol-core pathway and the limiting reactions involved in precursor synthesis.

## Conclusion

Metabolic flux through primary metabolism leads to the synthesis of several key intermediates necessary for the synthesis of the huge diversity of specialized compounds involved in fruit organoleptic and nutritional characteristics. Unfortunately, a limitation of primary metabolism engineering involves the lack of a correlation between primary metabolites pools and the levels of secondary metabolites that are derived from them. The emergence of new approaches like -omics (i.e., metabolomics and next-generation sequencing) technologies will allow us to combine multi-level transcriptional regulation and pathway rerouting to facilitate the metabolic engineering for fruit biofortification of these compounds. As described in this review, several approaches including crossing techniques and transformation could achieve the metabolic engineering to favor the accumulation of these specific beneficial metabolites derived from primary metabolism. In this sense, the use of genetically modified plants, overexpressing the genes responsible for the limiting steps in the interface between primary and secondary metabolism, is an essential tool, allowing a better understanding of the regulatory network that controls metabolic flux. Furthermore, the identification of natural allelic variation within germplasm is a powerful approach for fruit quality improvement in breeding programs, as it bypasses transgenic plant practice. Since obtaining high quality fruits has become one of the primary goals of breeding programs, application of these natural polymorphisms in marker-assisted selection can allow the rapid selection of varieties with enhanced organoleptic and nutritional characteristics and lead to greater economic returns for the industry.

## Author Contributions

All authors did the literature research, drafted the review, and helped write the final manuscript.

### Conflict of Interest Statement

The authors declare that the research was conducted in the absence of any commercial or financial relationships that could be construed as a potential conflict of interest.

## References

[ref1] Abbas F. Ke Y. Yu R. Yue Y. Amanullah S. Jahangir M. M.. (2017). Volatile terpenoids: multiple functions, biosynthesis, modulation and manipulation by genetic engineering. Planta 246, 803–816. 10.1007/s00425-017-2749-x, PMID: 28803364

[ref2] Aharoni A. Galili G. (2011). Metabolic engineering of the plant primary-secondary metabolism interface. Curr. Opin. Biotechnol. 22, 239–244. 10.1016/j.copbio.2010.11.004, PMID: 21144730

[ref3] Aharoni A. Giri A. P. Verstappen F. W. A. Bertea C. M. Sevenier R. Sun Z.. (2004). Gain and loss of fruit flavor compounds produced by wild and cultivated strawberry species. Plant Cell 16, 3110–3131. 10.1105/tpc.104.023895, PMID: 15522848 PMC527202

[ref4] Aharoni A. Keizer L. C. Bouwmeester H. J. Sun Z. Alvarez-Huerta M. Verhoeven H. A.. (2000). Identification of the SAAT gene involved in strawberry flavor biogenesis by use of DNA microarrays. Plant Cell 12, 647–662. 10.1105/tpc.12.5.647, PMID: 10810141 PMC139918

[ref5] Al-Babili S. Bouwmeester H. J. (2015). Strigolactones, a novel carotenoid-derived plant hormone. Annu. Rev. Plant Biol. 66, 161–186. 10.1146/annurev-arplant-043014-114759, PMID: 25621512

[ref6] Alós E. Cercós M. Rodrigo M. J. Zacarías L. Talón M. (2006). Regulation of color break in citrus fruits. Changes in pigment profiling and gene expression induced by gibberellins and nitrate, two ripening retardants. J. Agric. Food Chem. 54, 4888–4895. 10.1021/jf0606712, PMID: 16787044

[ref7] Araguez I. Osorio S. Hoffmann T. Rambla J. L. Medina-Escobar N. Granell A.. (2013). Eugenol production in achenes and receptacles of strawberry fruits is catalyzed by synthases exhibiting distinct kinetics. Plant Physiol. 163, 946–958. 10.1104/pp.113.224352, PMID: 23983228 PMC3793070

[ref8] Atkinson R. G. (2018). Phenylpropenes: occurrence, distribution, and biosynthesis in fruit. J. Agric. Food Chem. 66, 2259–2272. 10.1021/acs.jafc.6b04696, PMID: 28006900

[ref9] Basset G. J. Latimer S. Fatihi A. Soubeyrand E. Block A. (2017). Phylloquinone (vitamin K1): occurrence, biosynthesis and function. Mini Rev. Med. Chem. 17, 1028–1038. 10.2174/1389557516666160623082714, PMID: 27337968

[ref10] Basset G. Quinlivan E. Ravanel S. Rebeille F. (2004). Folate synthesis in plants: the p-aminobenzoate branch is initiated by a bifunctional PabA-PabB protein that is targeted to plastids. Proc. Natl. Acad. Sci. USA 101, 1–6. 10.1073/pnas.0308331100 14745019 PMC341757

[ref11] Battilana J. Costantini L. Emanuelli F. Sevini F. Segala C. Moser S.. (2009). The 1-deoxy-d-xylulose 5-phosphate synthase gene co-localizes with a major QTL affecting monoterpene content in grapevine. Theor. Appl. Genet. 118, 653–669. 10.1007/s00122-008-0927-8, PMID: 19037624

[ref12] Beauvoit B. Belouah I. Bertin N. Cakpo C. B. Colombié S. Dai Z.. (2018). Putting primary metabolism into perspective to obtain better fruits. Ann. Bot. 122, 1–21. 10.1093/aob/mcy057, PMID: 29718072 PMC6025238

[ref500] Beaudoin N. Rothstein S. J. (1997). Developmental regulation of two tomato lipoxygenase promoters in transgenic tobacco and tomato. Plant. Mol. Biol. 33, 835–846. PMID: 9106507 10.1023/a:1005773722657

[ref13] Beekwilder J. Alvarez-Huerta M. Neef E. Verstappen F. W. A. Bouwmeester H. J. Aharoni A. (2004). Functional characterization of enzymes forming volatile esters from strawberry and banana. Plant Physiol. 135, 1865–1878. 10.1104/pp.104.042580, PMID: 15326278 PMC520758

[ref14] Behnke K. Ehlting B. Teuber M. Bauerfeind M. Louis S. Hänsch R.. (2007). Transgenic, non-isoprene emitting poplars don’t like it hot. Plant J. 51, 485–499. 10.1111/j.1365-313X.2007.03157.x, PMID: 17587235

[ref15] Beltran J. C. M. Stange C. (2016). “Apocarotenoids: a new carotenoid-derived pathway” in Carotenoids in nature: Biosynthesis, regulation and function. ed. Stange C. (Cham: Springer International Publishing), 239–272.

[ref16] Bleeker P. M. Spyropoulou E. A. Diergaarde P. J. Volpin H. De Both M. T. J. Zerbe P.. (2011). RNA-seq discovery, functional characterization, and comparison of sesquiterpene synthases from *Solanum lycopersicum* and *Solanum habrochaites* trichomes. Plant Mol. Biol. 77, 323–336. 10.1007/s11103-011-9813-x, PMID: 21818683 PMC3193516

[ref17] Bogs J. (2005). Proanthocyanidin synthesis and expression of genes encoding leucoanthocyanidin reductase and anthocyanidin reductase in developing grape berries and grapevine leaves. Plant Physiol. 139, 652–663. 10.1104/pp.105.064238, PMID: 16169968 PMC1255985

[ref18] Bohlmann J. Meyer-Gauen G. Croteau R. (1998). Plant terpenoid synthases: molecular biology and phylogenetic analysis. Proc. Natl. Acad. Sci. USA 95, 4126–4133. 10.1073/pnas.95.8.4126 9539701 PMC22453

[ref19] Botella-Pavía P. Besumbes Ó. Phillips M. A. Carretero-Paulet L. Boronat A. Rodríguez-Concepción M. (2004). Regulation of carotenoid biosynthesis in plants: evidence for a key role of hydroxymethylbutenyl diphosphate reductase in controlling the supply of plastidial isoprenoid precursors. Plant J. 40, 188–199. 10.1111/j.1365-313X.2004.02198.x, PMID: 15447646

[ref20] Botella-Pavía P. Rodríguez-Concepción M. (2006). Carotenoid biotechnology in plants for nutritionally improved foods. Physiol. Plant. 126, 369–381. 10.1111/j.1399-3054.2006.00632.x

[ref21] Bouvier F. Harlingue A. Suire C. Backhaus R. A. Camara B. (1998). Dedicated roles of plastid transketolases during the early onset of isoprenoid biogenesis in pepper fruits 1. Plant Physiol. 117, 1423–1431. 10.1104/pp.117.4.1423, PMID: 9701598 PMC34906

[ref22] Butelli E. Titta L. Giorgio M. Mock H. P. Matros A. Peterek S.. (2008). Enrichment of tomato fruit with health-promoting anthocyanins by expression of select transcription factors. Nat. Biotechnol. 26, 1301–1308. 10.1038/nbt.1506, PMID: 18953354

[ref23] Buttery R. Teranishi R. Flath R. Ling L. (1989). “Fresh tomato volatiles – composition and sensory studies” in Flavour chemistry: Trends and developments. eds. Teranishi R. Buttery R. Shahidi F. (Washington, DC: American Chemical Society), 213–222.

[ref24] Carrington Y. Guo J. Fillo A. Kwon J. Tran L. T. Ehlting J. (2018). Evolution of a secondary metabolic pathway from primary metabolism: shikimate and quinate biosynthesis in plants. Plant J. 95, 823–833. 10.1111/tpj.13990, PMID: 29894016

[ref25] Cazzonelli C. I. Pogson B. J. (2010). Source to sink: regulation of carotenoid biosynthesis in plants. Trends Plant Sci. 15, 266–274. 10.1016/j.tplants.2010.02.003, PMID: 20303820

[ref26] Chaurasiya N. D. Sangwan N. S. Sabir F. Misra L. Sangwan R. S. (2012). Withanolide biosynthesis recruits both mevalonate and DOXP pathways of isoprenogenesis in Ashwagandha *Withania somnifera* L. (Dunal). Plant Cell Rep. 31, 1889–1897. 10.1007/s00299-012-1302-4, PMID: 22733207

[ref27] Chen G. Hackett R. Walker D. Taylor A. Lin Z. Grierson D. (2004). Identification of a specific isoform of tomato lipoxygenase (TomloxC) involved in the generation of fatty acid-derived flavor compounds. Plant Physiol. 136, 2641–2651. 10.1104/pp.104.041608, PMID: 15347800 PMC523329

[ref28] Contreras C. Tjellström H. Beaudry R. M. (2016). Relationships between free and esterified fatty acids and LOX-derived volatiles during ripening in apple. Postharvest Biol. Technol. 112, 105–113. 10.1016/j.postharvbio.2015.10.009

[ref29] Dal Cin V. Tieman D. M. Tohge T. McQuinn R. de Vos R. C. H. Osorio S.. (2011). Identification of genes in the phenylalanine metabolic pathway by ectopic expression of a MYB transcription factor in tomato fruit. Plant Cell 23, 2738–2753. 10.1105/tpc.111.086975, PMID: 21750236 PMC3226207

[ref30] Dalla Costa L. Emanuelli F. Trenti M. Moreno-Sanz P. Lorenzi S. Coller E. (2018). Induction of terpene biosynthesis in berries of microvine transformed with VvDXS1 alleles. Front. Plant Sci. 8, 1–14. 10.3389/fpls.2017.02244 PMC5776104 29387072

[ref31] de Kraker J.-W. Gershenzon J. (2011). From amino acid to glucosinolate biosynthesis: protein sequence changes in the evolution of methylthioalkylmalate synthase in *Arabidopsis*. Plant Cell 23, 38–53. 10.1105/tpc.110.079269, PMID: 21205930 PMC3051243

[ref32] Degenhardt J. Köllner T. G. Gershenzon J. (2009). Monoterpene and sesquiterpene synthases and the origin of terpene skeletal diversity in plants. Phytochemistry 70, 1621–1637. 10.1016/j.phytochem.2009.07.030, PMID: 19793600

[ref33] Deshpande A. B. Chidley H. G. Oak P. S. Pujari K. H. Giri A. P. Gupta V. S. (2017). Isolation and characterization of 9-lipoxygenase and epoxide hydrolase 2 genes: insight into lactone biosynthesis in mango fruit (*Mangifera indica* L.). Phytochemistry 138, 65–75. 10.1016/j.phytochem.2017.03.002, PMID: 28291596

[ref34] Díaz de la Garza R. Quinlivan E. P. Klaus S. M. J. Basset G. J. C. Gregory J. F. Hanson A. D. (2004). Folate biofortification in tomatoes by engineering the pteridine branch of folate synthesis. Proc. Natl. Acad. Sci. USA 101, 13720–13725. 10.1073/pnas.0404208101 15365185 PMC518823

[ref35] Duchêne E. Butterlin G. Claudel P. Dumas V. Jaegli N. Merdinoglu D. (2009). A grapevine (*Vitis vinifera* L.) deoxy-d-xylulose synthase gene colocates with a major quantitative trait loci for terpenol content. Theor. Appl. Genet. 118, 541–552. 10.1007/s00122-008-0919-8, PMID: 19002427

[ref36] Dudareva N. Andersson S. Orlova I. Gatto N. Reichelt M. Rhodes D. (2005). The nonmevalonate pathway supports both monoterpene and sesquiterpene formation in snapdragon flowers. Proc. Natl. Acad. Sci. USA 102, 933–938. 10.1007/BF02534636 15630092 PMC545543

[ref37] Dudareva N. Klempien A. Muhlemann J. K. Kaplan I. (2013). Biosynthesis, function and metabolic engineering of plant volatile organic compounds. New Phytol. 198, 16–32. 10.1111/nph.12145, PMID: 23383981

[ref38] Dudareva N. Pichersky E. (2008). Metabolic engineering of plant volatiles. Curr. Opin. Biotechnol. 19, 181–189. 10.1016/j.copbio.2008.02.011, PMID: 18394878

[ref39] Dudareva N. Pichersky E. Gershenzon J. (2004). Biochemistry of plant volatiles. Plant Physiol. 135, 1893–1902. 10.1104/pp.104.049981, PMID: 15326281 PMC520761

[ref40] Eisenreich W. Bacher A. Arigoni D. Rohdich F. (2004). Biosynthesis of isoprenoids *via* the non-mevalonate pathway. Cell. Mol. Life Sci. 61, 1401–1426. 10.1007/s00018-004-3381-z, PMID: 15197467 PMC11138651

[ref41] Emanuelli F. Battilana J. Costantini L. Le Cunff L. Boursiquot J.-M. This P.. (2010). A candidate gene association study on Muscat flavor in grapevine (*Vitis vinifera* L.). BMC Plant Biol. 10:241. 10.1186/1471-2229-10-241, PMID: 21062440 PMC3095323

[ref42] Enfissi E. M. A. Fraser P. D. Lois L. M. Boronat A. Schuch W. Bramley P. M. (2005). Metabolic engineering of the mevalonate and non-mevalonate isopentenyl diphosphate-forming pathways for the production of health-promoting isoprenoids in tomato. Plant Biotechnol. J. 3, 17–27. 10.1111/j.1467-7652.2004.00091.x, PMID: 17168896

[ref43] Eshed Y. Zamir D. (1995). An introgression line population of Lycopersicon pennellii in the cultivated tomato enables the identification and fine mapping of yield-associated QTL. Genetics 141, 1147–1162. PMID: . 8582620 10.1093/genetics/141.3.1147 PMC1206837

[ref44] Falara V. Akhtar T. A. Nguyen T. T. H. Spyropoulou E. A. Bleeker P. M. Schauvinhold I.. (2011). The tomato terpene synthase gene family. Plant Physiol. 157, 770–789. 10.1104/pp.111.179648, PMID: 21813655 PMC3192577

[ref45] Fanciullino A. Cercós M. A. C. Dhuique-Mayer C. Froelicher Y. Talón M. Ollitrault P.. (2008). Changes in carotenoid content and biosynthetic gene expression in juice sacs of four orange varieties (*Citrus sinensis*) differing in flesh fruit color. J. Agric. Food Chem. 56, 3628–3638. 10.1021/jf0732051, PMID: 18433104

[ref46] Fellman J. K. Miller T. W. Mattinson D. S. Mattheis J. P. (2000). Factors that influence biosynthesis of volatile flavor compounds in apple fruits. HortScience 35, 1026–1033. 10.21273/HORTSCI.35.6.1026

[ref47] Feng S. Niu L. Suh J. H. Hung W. L. Wang Y. (2018). Comprehensive metabolomics analysis of mandarins (*Citrus reticulata*) as a tool for variety, rootstock, and grove discrimination. J. Agric. Food Chem. 66, 10317–10326. 10.1021/acs.jafc.8b03877, PMID: 30205680

[ref48] Feussner I. Wasternack C. (2002). The lipoxygenase pathway. Annu. Rev. Plant Biol. 53, 275–297. 10.1146/annurev.arplant.53.100301.135248, PMID: 12221977

[ref49] Fiedor J. Burda K. (2014). Potential role of carotenoids as antioxidants in human health and disease. Nutrients 6, 466–488. 10.3390/nu6020466, PMID: 24473231 PMC3942711

[ref50] Fraser P. D. Bramley P. M. (2004). The biosynthesis and nutritional uses of carotenoids. Prog. Lipid Res. 43, 228–265. 10.1016/j.plipres.2003.10.002, PMID: 15003396

[ref51] Fraser C. M. Chapple C. (2011). The phenylpropanoid pathway in Arabidopsis. Arabidopsis Book 9:e0152. 10.1199/tab.0152 22303276 PMC3268504

[ref52] Garbowicz K. Liu Z. Alseekh S. Tieman D. Taylor M. Kuhalskaya A.. (2018). Quantitative trait loci analysis identifies a prominent gene involved in the production of fatty acid-derived flavor volatiles in tomato. Mol. Plant 11, 1147–1165. 10.1016/j.molp.2018.06.003, PMID: 29960108

[ref53] Giampieri F. Forbes-Hernandez T. Y. Gasparrini M. Alvarez-Suarez J. M. Afrin S. Bompadre S.. (2015). Strawberry as a health promoter: an evidence based review. Food Funct. 6, 1386–1398. 10.1039/C5FO00147A, PMID: 25803191

[ref54] Giovannoni J. (2004). Genetic regulation of fruit development and ripening. Plant Cell 16, 170–181. 10.1105/tpc.019158 PMC2643394 15010516

[ref55] Gonda I. Bar E. Portnoy V. Lev S. Burger J. Schaffer A. A.. (2010). Branched-chain and aromatic amino acid catabolism into aroma volatiles in *Cucumis melo* L. fruit. J. Exp. Bot. 61, 1111–1123. 10.1093/jxb/erp390, PMID: 20065117 PMC2826658

[ref56] Gonda I. Davidovich-Rikanati R. Bar E. Lev S. Jhirad P. Meshulam Y.. (2018). Differential metabolism of L-phenylalanine in the formation of aromatic volatiles in melon (*Cucumis melo* L.) fruit. Phytochemistry 148, 122–131. 10.1016/j.phytochem.2017.12.018, PMID: 29448137

[ref57] Gonda I. Lev S. Bar E. Sikron N. Portnoy V. Davidovich-Rikanati R.. (2013). Catabolism of L-methionine in the formation of sulfur and other volatiles in melon (*Cucumis melo* L.) fruit. Plant J. 74, 458–472. 10.1111/tpj.12149, PMID: 23402686

[ref58] Graziana A. Boudet A. M. (1980). 3-Deoxy-d-arabino heptulosonate 7-phosphate synthase from *Zea mays*: general properties and regulation by tryptophan. Plant Cell Physiol. 21, 793–802. 10.1093/oxfordjournals.pcp.a076054

[ref59] Gritsunov A. Peek J. Diaz Caballero J. Guttman D. Christendat D. (2018). Structural and biochemical approaches uncover multiple evolutionary trajectories of plant quinate dehydrogenases. Plant J. 95, 812–822. 10.1111/tpj.13989 29890023

[ref60] Gutensohn M. Orlova I. Nguyen T. T. H. Davidovich-Rikanati R. Ferruzzi M. G. Sitrit Y.. (2013). Cytosolic monoterpene biosynthesis is supported by plastid-generated geranyl diphosphate substrate in transgenic tomato fruits. Plant J. 75, 351–363. 10.1111/tpj.12212, PMID: 23607888

[ref61] Haffner T. Tressl R. (1998). Stereospecific metabolism of isomeric epoxyoctadecanoic acids in the lactone-producing yeast *Sporidiobolus salmonicolor*. Lipids 33, 47–58. 10.1007/s11745-998-0179-9, PMID: 9470173

[ref62] Hampel D. Mosandl A. Wüst M. (2006). Biosynthesis of mono- and sesquiterpenes in strawberry fruits and foliage: 2H labeling studies. J. Agric. Food Chem. 54, 1473–1478. 10.1021/jf0523972, PMID: 16478276

[ref63] Hannum S. M. (2004). Potential impact of strawberries on human health: a review of the science potential impact of strawberries on human health: a review. Crit. Rev. Food Sci. Nutr. 44, 1–17. 10.1080/10408690490263756, PMID: 15077879

[ref64] Hashimoto H. Uragami C. Cogdell R. J. (2016). “Carotenoids and photosynthesis” in Carotenoids in nature: Biosynthesis, regulation and function. ed. Stange C. (Cham: Springer International Publishing), 111–139.

[ref65] Hassan S. Mathesius U. (2012). The role of flavonoids in root-rhizosphere signalling: opportunities and challenges for improving plant-microbe interactions. J. Exp. Bot. 63, 3429–3444. 10.1093/jxb/err430, PMID: 22213816

[ref66] Hemmerlin A. Harwood J. L. Bach T. J. (2012). A raison d’être for two distinct pathways in the early steps of plant isoprenoid biosynthesis? Prog. Lipid Res. 51, 95–148. 10.1016/j.plipres.2011.12.001, PMID: 22197147

[ref67] Herrmann K. M. (1995). The shikimate pathway as an entry to aromatic secondary metabolism. Plant Physiol. 107, 7–12. 10.1104/pp.107.1.7, PMID: 7870841 PMC161158

[ref68] Hildebrand D. F. Rodriguez J. G. Legg C. S. Brown’ G. C. Bookjans G. (1989). The effects of wounding and mite infestation on soybean leaf lipoxygenase levels. Z. Naturforsch. C J. Biosci. 44, 655–659. 10.1515/znc-1989-7-818

[ref69] Hirschberg J. (2001). Carotenoid biosynthesis in flowering plants. Curr. Opin. Biotechnol. 4, 210–218. 10.1016/S1369-5266(00)00163-1 11312131

[ref70] Hossain T. Rosenberg I. Selhub J. Kishore G. Beachy R. Schubert K. (2004). Enhancement of folates in plants through metabolic engineering. Proc. Natl. Acad. Sci. USA 101, 5158–5163. 10.1073/pnas.0401342101 15044686 PMC387390

[ref71] Hou X. Rivers J. León P. McQuinn R. P. Pogson B. J. (2016). Synthesis and function of apocarotenoid signals in plants. Trends Plant Sci. 21, 792–803. 10.1016/j.tplants.2016.06.001, PMID: 27344539

[ref72] Ikoma Y. Matsumoto H. Kato M. (2016). Diversity in the carotenoid profiles and the expression of genes related to carotenoid accumulation among citrus genotypes. Breed. Sci. 66, 139–147. 10.1270/jsbbs.66.139, PMID: 27069398 PMC4780797

[ref73] Jäpelt R. B. Jakobsen J. (2016). Analysis of vitamin K1 in fruits and vegetables using accelerated solvent extraction and liquid chromatography tandem mass spectrometry with atmospheric pressure chemical ionization. Food Chem. 192, 402–408. 10.1016/j.foodchem.2015.06.111, PMID: 26304366

[ref74] Jetti R. R. Yang E. Kurnianta A. Finn C. Qian M. C. (2007). Quantification of selected aroma-active compounds in strawberries by headspace solid-phase microextraction gas chromatography and correlation with sensory descriptive analysis. J. Food Sci. 72, S487–S496. 10.1111/j.1750-3841.2007.00445.x 17995662

[ref75] Jiang Z. Kempinski C. Chappell J. (2016). Extraction and analysis of terpenes/terpenoids. Curr. Protoc. Plant Biol. 1, 345–358. 10.1002/cppb.20024, PMID: 27868090 PMC5113832

[ref76] Ju Y. L. Liu M. Zhao H. Meng J. F. Fang Y. L. (2016). Effect of exogenous abscisic acid and methyl jasmonate on anthocyanin composition, fatty acids, and volatile compounds of cabernet sauvignon (*Vitis vinifera* L.) grape berries. Molecules 21:1354. 10.3390/molecules21101354, PMID: 27754331 PMC6273220

[ref77] Kessler A. Halitschke R. Baldwin I. T. (2004). Silencing the jasmonate cascade: induced plant defenses and insect populations. Science 305, 665–668. 10.1126/science.1096931, PMID: 15232071

[ref78] Kim Y. K. Kim J. K. Kim Y. B. Lee S. Kim S. U. Park S. U. (2013). Enhanced accumulation of phytosterol and triterpene in hairy root cultures of *Platycodon grandiflorum* by overexpression of panax ginseng 3-hydroxy-3-methylglutaryl-coenzyme a reductase. J. Agric. Food Chem. 61, 1928–1934. 10.1021/jf304911t, PMID: 23298228

[ref79] Klee H. J. Tieman D. M. (2018). The genetics of fruit flavour preferences. Nat. Rev. Genet. 19, 347–356. 10.1038/s41576-018-0002-5, PMID: 29563555

[ref80] Kochevenko A. Araújo W. L. Maloney G. S. Tieman D. M. Do P. T. Taylor M. G.. (2012). Catabolism of branched chain amino acids supports respiration but not volatile synthesis in tomato fruits. Mol. Plant 5, 366–375. 10.1093/mp/ssr108, PMID: 22199237

[ref81] Korkina L. G. (2007). Phenylpropanoids as naturally occurring antioxidants: from plant defense to human health. Cell. Mol. Biol. 53, 15–25. 10.1170/T772, PMID: 17519109

[ref82] Kroymann J. (2011). Natural diversity and adaptation in plant secondary metabolism. Curr. Opin. Plant Biol. 14, 246–251. 10.1016/j.pbi.2011.03.021, PMID: 21514879

[ref83] La Camera S. Gouzerh G. Dhondt S. Hoffmann L. Fritig B. Legrand M.. (2004). Metabolic reprogramming in plant innate immunity: the contributions of phenylpropanoid and oxylipin pathways. Immunol. Rev. 198, 267–284. 10.1111/j.0105-2896.2004.0129.x, PMID: 15199968

[ref84] Landete J. M. (2011). Ellagitannins, ellagic acid and their derived metabolites: a review about source, metabolism, functions and health. Food Res. Int. 44, 1150–1160. 10.1016/j.foodres.2011.04.027

[ref85] Lange B. Ghassemian M. (2003). Genome organization in *Arabidopsis thaliana*: a survey for genes involved in isoprenoid and chlorophyll metabolism. Plant Mol. Biol. 51, 925–948. 10.1023/A:1023005504702, PMID: 12777052

[ref86] Laule O. Furholz A. Chang H.-S. Zhu T. Wang X. Heifetz P. B. (2003). Crosstalk between cytosolic and plastidial pathways of isoprenoid biosynthesis in *Arabidopsis thaliana*. Proc. Natl. Acad. Sci. USA 100, 6866–6871. 10.1073/pnas.1031755100 12748386 PMC164538

[ref87] Leitzmann C. (2016). Characteristics and health benefits of phytochemicals. Forsch. Komplementärmed. 23, 69–74. 10.1159/000444063, PMID: 27160996

[ref88] Li G. Jia H. Li J. Wang Q. Zhang M. Teng Y. (2014). Emission of volatile esters and transcription of ethylene- and aroma-related genes during ripening of “Pingxiangli” pear fruit (*Pyrus ussuriensis* maxim). Sci. Hortic. 170, 17–23. 10.1016/j.scienta.2014.03.004

[ref89] Li Y. Qi H. Jin Y. Tian X. Sui L. Qiu Y. (2016). Role of ethylene in biosynthetic pathway of related-aroma volatiles derived from amino acids in oriental sweet melons (*Cucumis melo* var. makuwa Makino). Sci. Hortic. 201, 24–35. 10.1016/j.scienta.2015.12.053

[ref90] Lichtenthaler H. K. (2010). “The non-mevalonate DOXP/MEP (deoxyxylulose 5-phosphate/methylerythritol 4-phosphate) pathway of chloroplast isoprenoid and pigment biosynthesis” in The chloroplast: Basics and applications. eds. Rebeiz C. A. Benning C. Bohnert H. J. Daniell H. Hoober J. K. Lichtenthaler H. K. (Dordrecht, Netherlands: Springer), 95–118.

[ref91] Liu J. Osbourn A. Ma P. (2015a). MYB transcription factors as regulators of phenylpropanoid metabolism in plants. Mol. Plant 8, 689–708. 10.1016/j.molp.2015.03.012 25840349

[ref92] Liu L. Shao Z. Zhang M. Wang Q. (2015b). Regulation of carotenoid metabolism in tomato. Mol. Plant 8, 28–39. 10.1016/j.molp.2014.11.006 25578270

[ref93] Lois L. M. Rodríguez-Concepción M. Gallego F. Campos N. Boronat A. (2000). Carotenoid biosynthesis during tomato fruit development: regulatory role of 1-deoxy-D-xylulose 5-phosphate synthase. Plant J. 22, 503–513. 10.1046/j.1365-313x.2000.00764.x, PMID: 10886770

[ref94] Loreto F. Dicke M. Schnitzler J. P. Turlings T. C. J. (2014). Plant volatiles and the environment. Plant Cell Environ. 37, 1905–1908. 10.1111/pce.12369, PMID: 24811745

[ref95] Luo J. Butelli E. Hill L. Parr A. Niggeweg R. Bailey P.. (2008). AtMYB12 regulates caffeoyl quinic acid and flavonol synthesis in tomato: expression in fruit results in very high levels of both types of polyphenol. Plant J. 56, 316–326. 10.1111/j.1365-313X.2008.03597.x, PMID: 18643978

[ref96] Maloney G. S. Kochevenko A. Tieman D. M. Tohge T. Krieger U. Zamir D.. (2010). Characterization of the branched-chain amino acid aminotransferase enzyme family in tomato. Plant Physiol. 153, 925–936. 10.1104/pp.110.154922, PMID: 20435740 PMC2899903

[ref97] Manach C. (2004). Polyphenols: food sources and bioavailability. Am. J. Clin. Nutr. 79, 727–747. 10.1093/ajcn/79.5.727, PMID: 15113710

[ref98] Manela N. Oliva M. Ovadia R. Sikron-Persi N. Ayenew B. Fait A. (2015). Phenylalanine and tyrosine levels are rate-limiting factors in production of health promoting metabolites in *Vitis vinifera* cv. Gamay red cell suspension. Front. Plant Sci. 6, 1–13. 10.3389/fpls.2015.00538 26236327 PMC4503893

[ref99] Manríquez D. El-Sharkawy I. Flores F. B. El-Yahyaoui F. Regad F. Bouzayen M.. (2006). Two highly divergent alcohol dehydrogenases of melon exhibit fruit ripening-specific expression and distinct biochemical characteristics. Plant Mol. Biol. 61, 675–685. 10.1007/s11103-006-0040-9, PMID: 16897483

[ref100] Markovic D. Colzi I. Taiti C. Ray S. Scalone R. Gregory Ali J.. (2019). Airborne signals synchronize the defenses of neighboring plants in response to touch. J. Exp. Bot. 70, 691–700. 10.1093/jxb/ery375, PMID: 30380091 PMC6322579

[ref101] Mwenda C. M. Matsui K. (2014). The importance of lipoxygenase control in the production of green leaf volatiles by lipase-dependent and independent pathways. Plant Biotechnol. 31, 445–452. 10.5511/plantbiotechnology.14.0924a

[ref102] Nambara E. Marion-Poll A. (2005). Abscisic acid biosynthesis and catabolism. Annu. Rev. Plant Biol. 56, 165–185. 10.1146/annurev.arplant.56.032604.144046, PMID: 15862093

[ref103] Negre-Zakharov F. Long M. Dudareva N. (2009). Floral scent and fruit aromas inspired by nature. (New York, NY: Springer).

[ref104] Niemetz R. Gross G. G. (2005). Enzymology of gallotannin and ellagitannin biosynthesis. Phytochemistry 66, 2001–2011. 10.1016/j.phytochem.2005.01.009, PMID: 16153405

[ref105] Nisar N. Li L. Lu S. Khin N. C. Pogson B. J. (2015). Carotenoid metabolism in plants. Mol. Plant 8, 68–82. 10.1016/j.molp.2014.12.007, PMID: 25578273

[ref106] Niyogi K. K. Truong T. B. (2013). Evolution of flexible non-photochemical quenching mechanisms that regulate light harvesting in oxygenic photosynthesis. Curr. Opin. Plant Biol. 16, 307–314. 10.1016/j.pbi.2013.03.011, PMID: 23583332

[ref107] Nuutinen T. (2018). Medicinal properties of terpenes found in *Cannabis sativa* and *Humulus lupulus*. Eur. J. Med. Chem. 157, 198–228. 10.1016/j.ejmech.2018.07.076, PMID: 30096653

[ref108] Orlova I. Nagegowda D. A. Kish C. M. Gutensohn M. Maeda H. Varbanova M. (2009). The small subunit of snapdragon geranyl diphosphate synthase modifies the chain length specificity of tobacco geranylgeranyl diphosphate synthase in planta. Plant Cell 21, 4002–4017. 10.1105/tpc.109.071282 20028839 PMC2814502

[ref109] Ossipov V. Salminen J. P. Ossipova S. Haukioja E. Pihlaja K. (2003). Gallic acid and hydrolysable tannins are formed in birch leaves from an intermediate compound of the shikimate pathway. Biochem. Syst. Ecol. 31, 3–16. 10.1016/S0305-1978(02)00081-9

[ref110] Paetzold H. Garms S. Bartram S. Wieczorek J. Urós-Gracia E. M. Rodríguez-Concepción M.. (2010). The isogene 1-deoxy-D-xylulose 5-phosphate synthase 2 controls isoprenoid profiles, precursor pathway allocation, and density of tomato trichomes. Mol. Plant 3, 904–916. 10.1093/mp/ssq032, PMID: 20591838

[ref111] Peng G. Wang C. Song S. Fu X. Azam M. Grierson D.. (2013). The role of 1-deoxy-d-xylulose-5-phosphate synthase and phytoene synthase gene family in citrus carotenoid accumulation. Plant Physiol. Biochem. 71, 67–76. 10.1016/j.plaphy.2013.06.031, PMID: 23883976

[ref112] Pérez A. G. Olías R. Sanz C. Olías J. M. (1996). Furanones in strawberries: evolution during ripening and postharvest shelf life. J. Agric. Food Chem. 44, 3620–3624. 10.1021/jf960099m

[ref113] Petrussa E. Braidot E. Zancani M. Peresson C. Bertolini A. Patui S.. (2013). Plant flavonoids-biosynthesis, transport and involvement in stress responses. Int. J. Mol. Sci. 14, 14950–14973. 10.3390/ijms140714950, PMID: 23867610 PMC3742282

[ref114] Pichersky E. Noel J. P. Dudareva N. (2006). Biosynthesis of plant volatiles: nature’s diversity and ingenuity. Science 311, 808–811. 10.1126/science.1118510, PMID: 16469917 PMC2861909

[ref115] Pino J. A. Quijano C. E. (2012). Study of the volatile compounds from plum (*Prunus domestica* L. cv. Horvin) and estimation of their contribution to the fruit aroma. Food Sci. Technol. 32, 76–83. 10.1590/S0101-20612012005000006

[ref116] Poiroux-Gonord F. Bidel L. P. R. Fanciullino A. L. Gautier H. Lauri-Lopez F. Urban L. (2010). Health benefits of vitamins and secondary metabolites of fruits and vegetables and prospects to increase their concentrations by agronomic approaches. J. Agric. Food Chem. 58, 12065–12082. 10.1021/jf1037745, PMID: 21067179

[ref117] Poiroux-Gonord F. Fanciullino A. L. Poggi I. Urban L. (2013). Carbohydrate control over carotenoid build-up is conditional on fruit ontogeny in clementine fruits. Physiol. Plant. 147, 417–431. 10.1111/j.1399-3054.2012.01672.x, PMID: 22882610

[ref118] Pryor W. A. Stahl W. Rock C. L. (2000). Beta carotene: from biochemistry to clinical trials. Nutr. Rev. 58, 39–53. 10.1111/j.1753-4887.2000.tb07810.x 10748608

[ref119] Qin G. Tao S. Zhang H. Huang W. Wu J. Xu Y.. (2014). Evolution of the aroma volatiles of pear fruits supplemented with fatty acid metabolic precursors. Molecules 19, 20183–20196. 10.3390/molecules191220183, PMID: 25474290 PMC6271835

[ref120] Quadrana L. Almeida J. Otaiza S. N. Duffy T. da Silva J. V. C. de Godoy F.. (2013). Transcriptional regulation of tocopherol biosynthesis in tomato. Plant Mol. Biol. 81, 309–325. 10.1007/s11103-012-0001-4, PMID: 23247837

[ref121] Raab T. López-Ráez J. A. Klein D. Caballero J. L. Moyano E. Schwab W.. (2006). FaQR, required for the biosynthesis of the strawberry flavor compound 4-hydroxy-2,5-dimethyl-3(2H)-furanone, encodes an enone oxidoreductase. Plant Cell 18, 1023–1037. 10.1105/tpc.105.039784, PMID: 16517758 PMC1425863

[ref122] Rambla J. L. Trapero-Mozos A. Diretto G. Rubio-Moraga A. Granell A. Gómez-Gómez L. (2016). Gene-metabolite networks of volatile metabolism in Airen and Tempranillo grape cultivars revealed a distinct mechanism of aroma bouquet production. Front. Plant Sci. 7:1619. 10.3389/fpls.2016.01619 27833635 PMC5082229

[ref123] Ramírez Rivera N. G. García-Salinas C. Aragão F. J. L. Díaz de la Garza R. I. (2016). Metabolic engineering of folate and its precursors in Mexican common bean (*Phaseolus vulgaris* L.). Plant Biotechnol. J. 14, 2021–2032. 10.1111/pbi.12561, PMID: 26997331 PMC5043471

[ref124] Rao A. V. Rao L. G. (2007). Carotenoids and human health. Pharmacol. Res. 55, 207–216. 10.1016/j.phrs.2007.01.012, PMID: 17349800

[ref125] Reinink M. Borstlap A. C. (1982). 3-Deoxy-d-arabino-heptulosonate 7-phosphate synthase from pea leaves: inhibition by L-tyrosine. Plant Sci. Lett. 26, 167–171. 10.1016/0304-4211(82)90088-8

[ref126] Ribéreau-Gayon P. Boidron J. N. Terrier A. (1975). Aroma of Muscat grape varieties. J. Agric. Food Chem. 23, 1042–1047. 10.1021/jf60202a050

[ref127] Richter A. Schaff C. Zhang Z. Lipka A. E. Tian F. Köllner T. G.. (2016). Characterization of biosynthetic pathways for the production of the volatile homoterpenes DMNT and TMTT in *Zea mays*. Plant Cell 28, 2651–2665. 10.1105/tpc.15.00919, PMID: 27662898 PMC5134970

[ref128] Rippert P. Matringe M. (2002). Molecular and biochemical characterization of an *Arabidopsis thaliana* arogenate dehydrogenase with two highly similar and active protein domains. Plant Mol. Biol. 48, 361–368. 10.1023/A:1014018926676, PMID: 11905963

[ref129] Rodríguez-Concepción M. (2010). Supply of precursors for carotenoid biosynthesis in plants. Arch. Biochem. Biophys. 504, 118–122. 10.1016/j.abb.2010.06.016, PMID: 20561506

[ref130] Rodríguez-Concepción M. Boronat A. (2002). Elucidation of the methylerythritol phosphate pathway for isoprenoid biosynthesis in bacteria and plastids. A metabolic milestone achieved through genomics. Plant Physiol. 130, 1079–1089. 10.1104/pp.007138, PMID: 12427975 PMC1540259

[ref131] Rodriguez-Mateos A. Heiss C. Borges G. Crozier A. (2014). Berry (poly)phenols and cardiovascular health. J. Agric. Food Chem. 62, 3842–3851. 10.1021/jf403757g, PMID: 24059851

[ref132] Ronen G. Cohen M. Zamir D. Hirschberg J. (1999). Regulation of carotenoid biosynthesis during tomato fruit development: expression of the gene for lycopene epsilon-cyclase is down-regulated during ripening and is elevated in the mutant Delta. Plant J. 17, 341–351. 10.1046/j.1365-313X.1999.00381.x, PMID: 10205893

[ref133] Rowan D. D. Allen J. M. Fielder S. Hunt M. B. (1999). Biosynthesis of straight-chain ester volatiles in red delicious and granny smith apples using deuterium-labeled precursors. J. Agric. Food Chem. 47, 2553–2562. 10.1021/jf9809028, PMID: 10552526

[ref134] Rowan D. D. Hunt M. B. Dimouro A. Alspach P. A. Weskett R. Volz R. K.. (2009). Profiling fruit volatiles in the progeny of a “Royal Gala” X “Granny Smith” apple (*Malus x domestica*) cross. J. Agric. Food Chem. 57, 7953–7961. 10.1021/jf901678v, PMID: 19691320

[ref135] Rubin J. L. Jensen R. A. (1985). Differentially regulated isozymes of 3-deoxy-d-arabino-heptulosonate-7-phosphate synthase from seedlings of *Vigna radiata* [L.] Wilczek. Plant Physiol. 79, 711–718. 10.1104/pp.79.3.711, PMID: 16664478 PMC1074957

[ref136] Ruiz-Sola M. Á. Rodríguez-Concepción M. (2012). Carotenoid biosynthesis in *Arabidopsis*: a colorful pathway. Arabidopsis Book 10:e0158. 10.1199/tab.0158, PMID: 22582030 PMC3350171

[ref137] Saladié M. Wright L. P. Garcia-Mas J. Rodriguez-Concepcion M. Phillips M. A. (2014). The 2-C-methylerythritol 4-phosphate pathway in melon is regulated by specialized isoforms for the first and last steps. J. Exp. Bot. 65, 5077–5092. 10.1093/jxb/eru275, PMID: 25013119 PMC4144782

[ref138] Saxena S. P. Israels E. D. Israels L. G. (2001). Novel vitamin K-dependent pathways regulating cell survival. Apoptosis 6, 57–68. 10.1023/A:1009624111275, PMID: 11321042

[ref139] Schauer N. Semel Y. Roessner U. Gur A. Balbo I. Carrari F.. (2006). Comprehensive metabolic profiling and phenotyping of interspecific introgression lines for tomato improvement. Nat. Biotechnol. 24, 447–454. 10.1038/nbt1192, PMID: 16531992

[ref140] Schwab W. Davidovich-Rikanati R. Lewinsohn E. (2008). Biosynthesis of plant-derived flavor compounds. Plant J. 54, 712–732. 10.1111/j.1365-313X.2008.03446.x, PMID: 18476874

[ref141] Shen J. Tieman D. Jones J. B. Taylor M. G. Schmelz E. Huffaker A.. (2014). A 13-lipoxygenase, TomloxC, is essential for synthesis of C5 flavour volatiles in tomato. J. Exp. Bot. 65, 419–428. 10.1093/jxb/ert382, PMID: 24453226 PMC3904703

[ref142] Simpson K. Quiroz L. F. Rodriguez-Concepción M. Stange C. R. (2016). Differential contribution of the first two enzymes of the MEP pathway to the supply of metabolic precursors for carotenoid and chlorophyll biosynthesis in carrot (*Daucus carota*). Front. Plant Sci. 7, 1–10. 10.3389/fpls.2016.01344 27630663 PMC5005961

[ref143] Singh R. K. Ali S. A. Nath P. Sane V. A. (2011). Activation of ethylene-responsive p-hydroxyphenylpyruvate dioxygenase leads to increased tocopherol levels during ripening in mango. J. Exp. Bot. 62, 3375–3385. 10.1093/jxb/err006, PMID: 21430290 PMC3130165

[ref144] Storozhenko S. De Brouwer V. Volckaert M. Navarrete O. Blancquaert D. Zhang G. F.. (2007). Folate fortification of rice by metabolic engineering. Nat. Biotechnol. 25, 1277–1279. 10.1038/nbt1351, PMID: 17934451

[ref145] Stracke R. Werber M. Weisshaar B. (2001). The R2R3-MYB gene family in *Arabidopsis thaliana*. Curr. Opin. Plant Biol. 4, 447–456. 10.1016/S1369-5266(00)00199-0, PMID: 11597504

[ref146] Sun T. Yuan H. Cao H. Yazdani M. Tadmor Y. Li L. (2018). Carotenoid metabolism in plants: the role of plastids. Mol. Plant 11, 58–74. 10.1016/j.molp.2017.09.010, PMID: 28958604

[ref147] Sweetlove L. J. Nielsen J. Fernie A. R. (2017). Engineering central metabolism – a grand challenge for plant biologists. Plant J. 90, 749–763. 10.1111/tpj.13464, PMID: 28004455

[ref148] Tang Y. Zhang C. Cao S. Wang X. Qi H. (2015). The effect of CmLOXs on the production of volatile organic compounds in four aroma types of melon (*Cucumis melo*). PLoS One 10, 1–18. 10.1371/journal.pone.0143567 PMC4657985 26599669

[ref149] Tholl D. Kish C. M. Orlova I. Sherman D. Pichersky E. Dudareva N. (2015). Formation of monoterpenes in *Antirrhinum majus* and *Clarkia breweri* flowers involves heterodimeric geranyl diphosphate synthases. Plant Cell 16, 977–992. 10.1105/tpc.020156 PMC412871 15031409

[ref150] Tieman D. M. Loucas H. M. Kim J. Y. Clark D. G. Klee H. J. (2007). Tomato phenylacetaldehyde reductases catalyze the last step in the synthesis of the aroma volatile 2-phenylethanol. Phytochemistry 68, 2660–2669. 10.1016/j.phytochem.2007.06.005, PMID: 17644147

[ref151] Tieman D. Taylor M. Schauer N. Fernie A. R. Hanson A. D. Klee H. J. (2006). Tomato aromatic amino acid decarboxylases participate in synthesis of the flavor volatiles 2-phenylethanol and 2-phenylacetaldehyde. Proc. Natl. Acad. Sci. USA 103, 8287–8292. 10.1073/pnas.0602469103 16698923 PMC1472464

[ref152] Tieman D. Zhu G. Resende M. F. R. Lin T. Nguyen C. Bies D.. (2017). A chemical genetic roadmap to improved tomato flavor. Science 355, 391–394. 10.1126/science.aal1556, PMID: 28126817

[ref153] Ties P. Barringer S. (2012). Influence of lipid content and lipoxygenase on flavor volatiles in the tomato peel and flesh. J. Food Sci. 77, 830–837. 10.1111/j.1750-3841.2012.02775.x 22757705

[ref154] Tikunov Y. (2005). A novel approach for nontargeted data analysis for metabolomics. Large-scale profiling of tomato fruit volatiles. Plant Physiol. 139, 1125–1137. 10.1104/pp.105.068130, PMID: 16286451 PMC1283752

[ref155] Tokitomo Y. Steinhaus M. Büttner A. (2005). Odor-active constituents in fresh pineapple (*Ananas comosus* [L.] Merr.) by quantitative and sensory evaluation by quantitative and sensory evaluation. Biosci. Biotechnol. Biochem. 69, 1323–1330. 10.1271/bbb.69.1323 16041138

[ref156] Tokuriki N. Jackson C. J. Afriat-Jurnou L. Wyganowski K. T. Tang R. Tawfik D. S. (2012). Diminishing returns and tradeoffs constrain the laboratory optimization of an enzyme. Nat. Commun. 3, 1257–1259. 10.1038/ncomms2246 23212386

[ref157] Tzin V. Fernandez-Pozo N. Richter A. Schmelz E. A. Schoettner M. Schäfer M. (2015a). Dynamic maize responses to aphid feeding are revealed by a time series of transcriptomic and metabolomic assays. Plant Physiol. 169, 1727–1743. 10.1104/pp.15.01039 26378100 PMC4634079

[ref158] Tzin V. Malitsky S. Aharoni A. Galili G. (2009). Expression of a bacterial bi-functional chorismate mutase/prephenate dehydratase modulates primary and secondary metabolism associated with aromatic amino acids in Arabidopsis. Plant J. 60, 156–167. 10.1111/j.1365-313X.2009.03945.x, PMID: 19508381

[ref159] Tzin V. Malitsky S. Zvi M. Ben M. Bedair M. Sumner L.. (2012). Expression of a bacterial feedback-insensitive 3-deoxy-d-arabino-heptulosonate 7-phosphate synthase of the shikimate pathway in Arabidopsis elucidates potential metabolic bottlenecks between primary and secondary metabolism. New Phytol. 194, 430–439. 10.1111/j.1469-8137.2012.04052.x, PMID: 22296303

[ref160] Tzin V. Rogachev I. Meir S. Moyal Ben Zvi M. Masci T. Vainstein A. (2015b). Altered levels of aroma and volatiles by metabolic engineering of shikimate pathway genes in tomato fruits. AIMS Bioeng. 2, 75–92. 10.3934/bioeng.2015.2.75

[ref161] Tzin V. Rogachev I. Meir S. Zvi M. Ben M. Masci T.. (2013). Tomato fruits expressing a bacterial feedback-insensitive 3-deoxy-D-arabino-heptulosonate 7-phosphate synthase of the shikimate pathway possess enhanced levels of multiple specialized metabolites and upgraded aroma. J. Exp. Bot. 64, 4441–4452. 10.1093/jxb/ert250, PMID: 24006429 PMC3808321

[ref162] Ul-Hassan M. N. Zainal Z. Ismail I. (2015). Green leaf volatiles: biosynthesis, biological functions and their applications in biotechnology. Plant Biotechnol. J. 13, 727–739. 10.1111/pbi.12368, PMID: 25865366

[ref163] Vallarino J. G. de Abreu E Lima F. Soria C. Tong H. Pott D. M. Willmitzer L. (2018). Genetic diversity of strawberry germplasm using metabolomic biomarkers. Sci. Rep. 8:14386. 10.1038/s41598-018-32212-9 30258188 PMC6158285

[ref164] Vallarino J. G. Pott D. M. Cruz-rus E. Miranda L. Medina-minguez J. J. Valpuesta V.. (2019). Identification of quantitative trait loci and candidate genes for primary metabolite content in strawberry fruit. Hortic. Res. 6:4. 10.1038/s41438-018-0077-3, PMID: 30603090 PMC6312544

[ref165] Verberne M. C. Sansuk K. Bol J. F. Linthorst H. J. M. Verpoorte R. (2007). Vitamin K1 accumulation in tobacco plants overexpressing bacterial genes involved in the biosynthesis of salicylic acid. J. Biotechnol. 128, 72–79. 10.1016/j.jbiotec.2006.09.005, PMID: 17084477

[ref166] Verdonk J. C. (2005). ODORANT1 regulates fragrance biosynthesis in petunia flowers. Plant Cell 17, 1612–1624. 10.1105/tpc.104.028837, PMID: 15805488 PMC1091778

[ref167] Vivaldo G. Masi E. Taiti C. Caldarelli G. Mancuso S. (2017). The network of plants volatile organic compounds. Sci. Rep. 7, 1–18. 10.1038/s41598-017-10975-x 28887468 PMC5591229

[ref168] Vogt T. (2010). Phenylpropanoid biosynthesis. Mol. Plant 3, 2–20. 10.1093/mp/ssp106, PMID: 20035037

[ref169] Vogt J. Schiller D. Ulrich D. Schwab W. Dunemann F. (2013). Identification of lipoxygenase (LOX) genes putatively involved in fruit flavour formation in apple (*Malus × domestica*). Tree Genet. Genomes 9, 1493–1511. 10.1007/s11295-013-0653-5

[ref170] Vranová E. Coman D. Gruissem W. (2012). Structure and dynamics of the isoprenoid pathway network. Mol. Plant 5, 318–333. 10.1093/mp/sss015, PMID: 22442388

[ref171] Wang Y. Hossain D. Perry P. L. Adams B. Lin J. (2012). Characterization of volatile and aroma-impact compounds in persimmon (*Diospyros kaki* L., var. triumph) fruit by GC-MS and GC-O analyses. Flavour Fragr. J. 27, 141–148. 10.1002/ffj.2094

[ref172] Wang M. Zhang L. Boo K. H. Park E. Drakakaki G. Zakharov F. (2019). PDC1, a pyruvate/α-ketoacid decarboxylase, is involved in acetaldehyde, propanal and pentanal biosynthesis in melon (*Cucumis melo* L.) fruit. Plant J. 98, 112–125. 10.1111/tpj.14204, PMID: 30556202

[ref173] Watanabe S. Ohtani Y. Tatsukami Y. Aoki W. Amemiya T. Sukekiyo Y.. (2017). Folate biofortification in hydroponically cultivated spinach by the addition of phenylalanine. J. Agric. Food Chem. 65, 4605–4610. 10.1021/acs.jafc.7b01375, PMID: 28548831

[ref174] Weiss E. A. (1997). Essential oil crops. (Wallingford, UK: CAB Intern).

[ref175] Weng J. K. (2013). The evolutionary paths towards complexity: a metabolic perspective. New Phytol. 201, 1141–1149. 10.1111/nph.12416 23889087

[ref176] Weng J. K. Philippe R. N. Noel J. P. (2012). The rise of chemodiversity in plants. Science 336, 1667–1670. 10.1126/science.1217411, PMID: 22745420

[ref177] Wink M. (2010). “Introduction: biochemistry, physiology and ecological functions of secondary metabolites” in Annual plant reviews: Biochemistry of plant secondary metabolism. ed. Wink M., Vol. 40 (Wiley-Blackwell), 1–19.

[ref178] Wolak N. Zawrotniak M. Gogol M. Kozik A. Rapala-Kozik M. (2017). Vitamins B1, B2, B3 and B9 – occurrence, biosynthesis pathways and functions in human nutrition. Mini Rev. Med. Chem. 17, 1075–1111. 10.2174/1389557516666160725095729, PMID: 27457213

[ref179] Wright L. P. Rohwer J. M. Ghirardo A. Hammerbacher A. Ortiz-Alcaide M. Raguschke B.. (2014). Deoxyxylulose 5-phosphate synthase controls flux through the methylerythritol 4-phosphate pathway in Arabidopsis. Plant Physiol. 165, 1488–1504. 10.1104/pp.114.245191, PMID: 24987018 PMC4119033

[ref180] Wu S. Schalk M. Clark A. Miles R. B. Coates R. Chappell J. (2006). Redirection of cytosolic or plastidic isoprenoid precursors elevates terpene production in plants. Nat. Biotechnol. 24, 1441–1447. 10.1038/nbt1251, PMID: 17057703

[ref181] Xie Q. Liu Z. Meir S. Rogachev I. Aharoni A. Klee H. J.. (2016). Altered metabolite accumulation in tomato fruits by coexpressing a feedback-insensitive AroG and the PhODO1 MYB-type transcription factor. Plant Biotechnol. J. 14, 2300–2309. 10.1111/pbi.12583, PMID: 27185473 PMC5103220

[ref182] Yan J. W. Ban Z. J. Lu H. Y. Li D. Poverenov E. Luo Z. S.. (2018). The aroma volatile repertoire in strawberry fruit: a review. J. Sci. Food Agric. 98, 4395–4402. 10.1002/jsfa.9039, PMID: 29603275

[ref183] Yan L. Zhai Q. Wei J. Li S. Wang B. Huang T. (2013). Role of tomato lipoxygenase D in wound-induced jasmonate biosynthesis and plant immunity to insect herbivores. PLoS Genet. 9:e1003964. 10.1371/journal.pgen.1003964 24348260 PMC3861047

[ref184] Yauk Y. K. Souleyre E. J. F. Matich A. J. Chen X. Wang M. Y. Plunkett B.. (2017). Alcohol acyl transferase 1 links two distinct volatile pathways that produce esters and phenylpropenes in apple fruit. Plant J. 91, 292–305. 10.1111/tpj.13564, PMID: 28380280

[ref185] Zhang Y. Butelli E. Alseekh S. Tohge T. Rallapalli G. Luo J. (2015). Multi-level engineering facilitates the production of phenylpropanoid compounds in tomato. Nat. Commun. 6, 1–11. 10.1038/ncomms9635 PMC4639801 26497596

[ref186] Zhang C. Cao S. Jin Y. Ju L. Chen Q. Xing Q. (2017a). Melon13-lipoxygenase CmLOX18 may be involved in C6 volatiles biosynthesis in fruit. Sci. Rep. 7, 1–12. 10.1038/s41598-017-02559-6 28588227 PMC5460189

[ref187] Zhang L. Li H. Gao L. Qi Y. Fu W. Li X. (2017b). Acyl-CoA oxidase 1 is involved in γ-decalactone release from peach (*Prunus persica*) fruit. Plant Cell Rep. 36, 829–842. 10.1007/s00299-017-2113-4 28238071

[ref188] Zhang B. Yin X. R. Li X. Yang S. L. Ferguson I. B. Chen K. S. (2009). Lipoxygenase gene expression in ripening kiwifruit in relation to ethyiene and aroma production. J. Agric. Food Chem. 57, 2875–2881. 10.1021/jf9000378, PMID: 19334761

[ref189] Zvi M. Ben M. Shklarman E. Masci T. Kalev H. Debener T.. (2012). PAP1 transcription factor enhances production of phenylpropanoid and terpenoid scent compounds in rose flowers. New Phytol. 195, 335–345. 10.1111/j.1469-8137.2012.04161.x, PMID: 22548501

